# Metabolomics insights into chronic kidney disease and modulatory effect of rhubarb against tubulointerstitial fibrosis

**DOI:** 10.1038/srep14472

**Published:** 2015-09-28

**Authors:** Zhi-Hao Zhang, Feng Wei, Nosratola D. Vaziri, Xian-Long Cheng, Xu Bai, Rui-Chao Lin, Ying-Yong Zhao

**Affiliations:** 1 Key Laboratory of Resource Biology and Biotechnology in Western China, Ministry of Education, the College of Life Sciences, Northwest University, No. 229 Taibai North Road, Xi’an, Shaanxi 710069, China; 2 Division of Nephrology and Hypertension, School of Medicine, University of California, Irvine, MedSci 1, C352, UCI Campus, Irvine, California, 92697, USA; 3 National Center for Natural Products Research, Department of BioMolecular Sciences, School of Pharmacy, University of Mississippi, Oxford, Mississippi, 38677, USA; 4 National Institutes for Food and Drug Control, State Food and Drug Administration, No. 2 Tiantan Xili, Beijing, 100050, China; 5 School of Chinese Materia Medica, Beijing University of Chinese Medicine, No. 11 North Third Ring Road, Beijing 100029, China; 6 Solution Centre, Waters Technologies (Shanghai) Ltd., No. 1000 Jinhai Road, Shanghai 201203, China

## Abstract

Chronic kidney disease (CKD) is a major public health problem worldwide. Rhubarb has been shown to have nephroprotective and anti-fibrotic activities in patients with CKD. However, bioactive fractions and biochemical mechanism of anti-fibrotic properties of rhubarb remain unclear. Here we applied ultra-performance liquid chromatography-quadrupole time-of-flight mass spectrometry together with univariate and multivariate statistical analyses to investigate the urinary metabolite profile in rats with adenine-induced CKD treated with the petroleum ether (PE)-, ethyl acetate (EA)- and n-butanol (BU)- extracts of rhubarb. Significant differences in renal function, kidney histopathology as well as metabolic profiles were observed between CKD and control rats. Changes in these parameters reflected characteristic phenotypes of CKD rats. We further identified a series of differential urinary metabolites for CKD rats, suggesting metabolic dysfunction in pathway of amino acid, purine, taurine, and choline metabolisms. Treatment with EA, BU and PE extracts of rhubarb improved renal function and histopathological abnormalities including interstitial fibrosis and inflammation, and either fully or partially reversed the abnormalities of the urinary metabolites. Among them, the nephroprotective effect of EA extract was stronger than BU and PE extracts. This work provides important mechanistic insights into the CKD and nephroprotective effects of different rhubarb extract against tubulo-interstitial fibrosis.

Chronic kidney disease (CKD) is a worldwide public health problem affecting approximately 8–10% of the population in Western countries^1^,^2^. CKD results in progressive loss of kidney function over periods of months to years. Currently, survival of patient with end-stage renal disease or chronic renal failure depends on dialysis therapy and renal transplantation which can cause enormous psychological and physical pain as well as high economic burden. Progression of CKD is associated with and largely mediated by glomerular and tubulo-interstitial fibrosis. Pathological mechanism of renal interstitial fibrosis is characterized by monocyte and macrophage infiltration, tubular atrophy and fibroblast proliferation/differentiation, extracellular matrix accumulation and tubulo-interstitial fibrosis^3^. Therefore, strategies aimed at blocking or retarding the glomerular and interstitial fibrosis can be highly effective in retarding CKD progression. Although numerous studies have explored therapeutic strategies to retard tubulo-interstitial fibrosis, effective anti-fibrotic treatment remains elusive.

Natural medicines or alternative medicines have been used and proven effective in the treatment of different diseases for thousands of years. Based on their theoretical therapeutic efficacy and long and wide clinical applications the natural anti-fibrotic medicines have gained increasing attention for prevention and treatment of CKD in Asia, Europe and North America. The key problem is that the efficacy of natural medicines has not been fully recognized due to the limited scientific data on the nature and mechanisms of their actions.

Rhubarb is a well-known natural medicine which is prepared from the dried roots of *Rheum tanguticum* Maxim ex Balf., *Rheum palmatum* L. or *Rheum officinale* Baill. (Polygonaceae). It has various pharmacological effects including diuretic, purgative, anti-inflammatory, nephroprotective, anti-tumor and anti-bacterial activities^4^. Rhubarb contains several different active components including anthraquinones, dianthrones, stilbenes, anthocyanins, flavonoids and polyphenols^5^. Treatment with Rhubarb has shown favorable effects in humans and animal with CKD^6^,^7^,^8^,^9^. However the biochemical mechanism of action and biological targets of rhubarb and its different components have not been fully elucidated.

Various components of the natural medicines can simultaneously hit different targets involved in the pathogenesis of the diseases. Untargeted metabolomic approach provides valuable information on global metabolic networks which is helpful in elucidating the underlying pathophysiological processes, clinical diagnosis, and the mechanisms of action and the response to the drug therapy^10^. In light of the holistic vision of multiple components and multiple targets of natural medicines, metabolomics has been widely applied to evaluate therapeutic effects of natural medicines^11^. Nuclear magnetic resonance (NMR) spectroscopy and mass spectrometry (MS) are two analytical tools commonly used in the assessment of metabolomics in CKD. The findings have demonstrated that progressive CKD is associated with changes in energy metabolism, amino acid metabolism, lipid metabolism and gut microbial metabolism^12^. In the MS-based metabolomics, ultra-performance liquid chromatography-quadrupole time-of-flight high-definition mass spectrometry (UPLC-QTOF/HDMS) is regarded to be suitable for untargeted metabonomics due to the high analytic speed and sensitivity and high resolution of chromatographic peaks for complex biological samples^13^,^14^. Our previous studies have demonstrated that UPLC-QTOF/HDMS-based metabolomic approach is a powerful tool to investigate pathogenesis of CKD^15^,^16^,^17^,^18^,^19^. Here, the UPLC-QTOF/HDMS-based urinary metabolomics was applied to investigate the urinary metabolite profile and potential biomarkers of adenine-induced CKD and the nephro-protective effect of three rhubarb extracts with different polarities including petroleum ether extract (PE), ethyl acetate extract (EA), and n-butanol extract (BU) (polarity: BU > EA > PE) in rats with adenine-induced CKD. The study further sought to identify the bioactive fractions of rhubarb and the mechanisms of their anti-fibrotic actions in this model.

## Results

### Biochemical Parameters results

**Figure 1:**
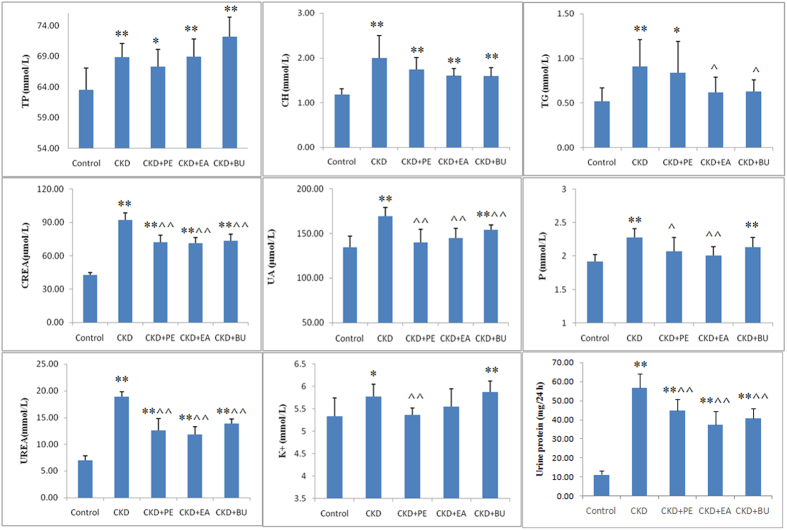
Biochemical parameters in the normal control, CKD+PE, CKD+EA and CKD+BU groups. Serum total protein (TP), cholesterol (CH), triglyceride (TG), creatinine (CREA), urea, uric acid (UA), phosphorus (P), potassium (K^+^) and urine protein in the control, untreated CKD, EA-treated CKD (CKD+EA), BU-treated CKD (CKD+BU) and PE-treated CKD (CKD+PE) groups. *p < 0.05, **p < 0.01 compared to control group; ^p < 0.05, ^^p < 0.01 compared to CKD group to control group; ^p < 0.05, ^^p < 0.01 compared to CKD group.

In the beginning, we compared the creatinine (CREA) and urea concentrations among control, CKD, CKD+Rub, CKD+PE, CKD+EA and CKD+BU groups (Fig. S1). CREA and urea concentrations were significantly increased in CKD group. The magnitude of increase in urea and CREA concentrations in CKD+Rub, CKD+PE, CKD+EA and CKD+BU groups was significantly less than those found in the untreated CKD rats. Furthermore, the magnitude of increase in urea and CREA concentrations in CKD+Rub was more than those found in the CKD+PE, CKD+EA and CKD+BU groups. Afterwards, we further focused on studying more biochemical parameters alterations among CKD+PE, CKD+EA and CKD+BU groups. As shown in Fig. 1, serum total protein (TP), cholesterol (CH), triglyceride (TG), CREA, urea, uric acid (UA), phosphorus (P), and potassium (K^+^) concentrations and urine protein excretion were significantly increased in CKD group compared to those found in the control group. However, compared with the control group, the EA-treated (EA+CKD) group showed no significant change in serum TG, UA, P or K^+^ concentrations. In addition the magnitude of rise in serum urea and CREA concentrations and urine protein excretion in the EA+CKD group was significantly less than that found in the untreated CKD group. Compared to the control group the PE-treated CKD (PE+CKD) group showed no significant change in serum UA, P and K^+^ concentrations. In addition the magnitude of increase in urea, CREA concentrations and urine protein excretion was significantly less than those found in the untreated CKD rats. Finally the BU-treated CKD (BU+CKD) group showed normal serum TG concentration and significantly lower increase in serum urea, CREA, and UA concentrations and urine protein excretion.

### Histological Data

**Figure 2:**
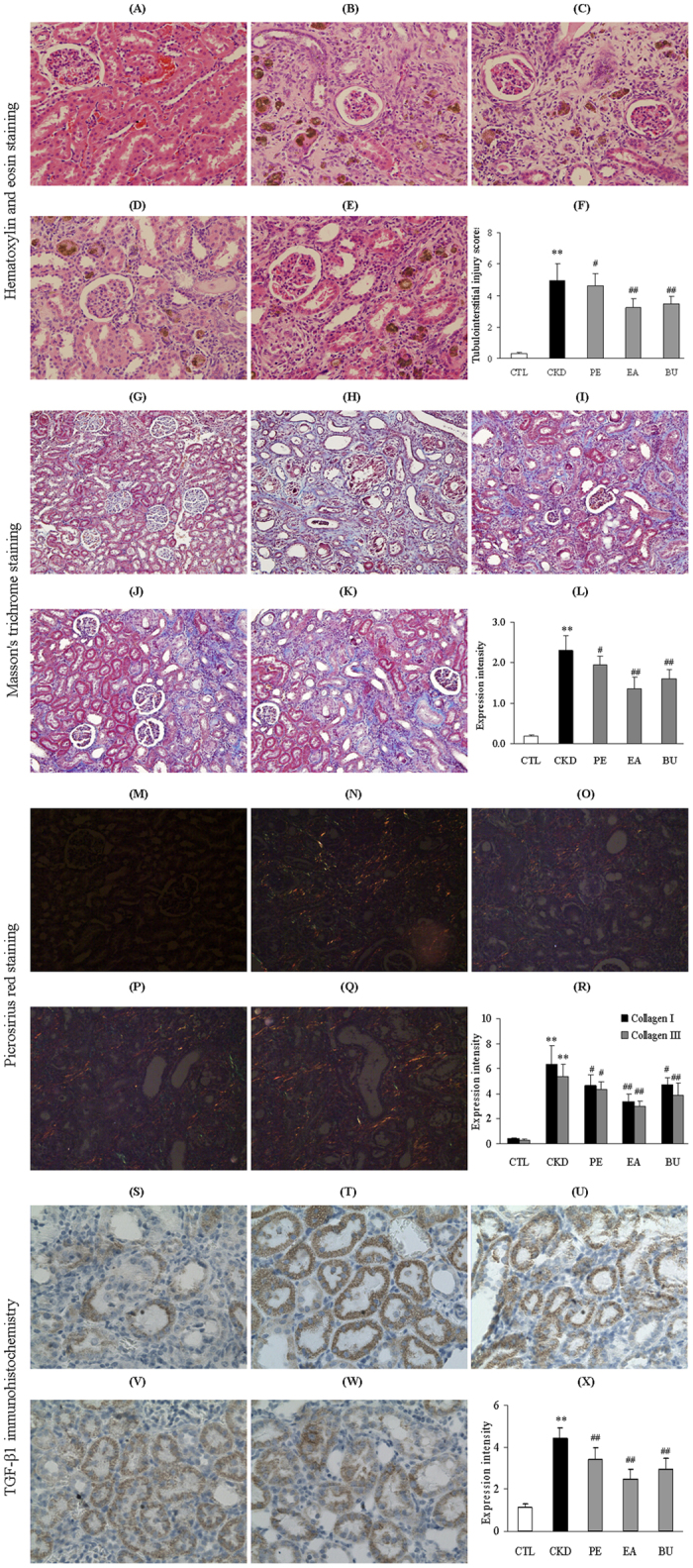
Representative photomicrographs of the H&E staining, Masson’s trichrome staining, Picrosirius red staining and TGF-β1 immunohistochemistry from kidney sections in normal control rat, CKD rat, CKD+PE rat, CKD+EA rat and CKD+BU rat at week 6. The kidney tissue in the CKD animals exhibited significant tubulointerstitial injury, increased collagen I, collagen III and fibrosis and heavy inflammatory cell infiltration which were significantly improved with PE, EA and BU extracts of rhubarb. Bar graphs depicting tubulointerstitial injury scores (**F**) and expression intensity of Masson’s staining (**L**), picrosirius red staining (**R**) and TGF-β1 immunohistochemistry intensity (**X**) in the study groups. (**A**,**G**,**M**,**S**) control rat; (**B**,**H**,**N**,**T**) CKD rat; (**C**,**I**,**O**,**U**) CKD+PE rat; (**D**,**J**,**P**,**V**) CKD+EA rat; (**E**,**K**,**Q**,**W**) CKD+BU rat. *p < 0.05, **p < 0.01 compared to control rat; ^#^p < 0.05, ^##^p < 0.01 compared to CKD rat.

Figure 2 shows representative photomicrographs of the hematoxylin and eosin (H&E) and Masson’s trichrome staining of the kidney tissues from the control, CKD, CKD+EA, CKD+BU and CKD+PE groups. Kidneys from the untreated CKD rats exhibited severe inflammatory cell infiltration, marked tubular dilation and interstitial fibrosis which were significantly improved in the rats treated with EA, BU and PE extracts of rhubarb. In order to quantify the severity of fibrosis, expressions of types I and III collagen were evaluated with picrosirius red staining (Fig. 2). Expression of types I and III collagen was significantly up-regulated in the untreated CKD group and markedly attenuated in the CKD+EA, CKD+BU and CKD+PE groups.

### TGF-β1 immunohistochemistry

TGF-β1 is a secreted protein which plays a major role in promoting fibrosis and regulation of many cellular functions, including the control of cell growth, cell proliferation, cell differentiation and apoptosis. Compared with the control group, TGF-β1 protein expression was significantly up-regulated in the untreated CKD rats, pointing to activation of fibrotic pathway. CKD-induced upregulation of renal tissue TGF-β1 was significantly attenuated in the CKD+EA, CKD+BU and CKD+PE groups (Fig. 2).

### Validation of UPLC−MS Conditions

The repeatability and precision were validated by the reduplicate analysis of six injections of the same quality control samples and six parallel samples prepared using the same preparation and method, respectively. The RSD of peak area and retention time were below 2.9% and 0.39% respectively and the reproducibility and precision were satisfactory for metabolomic analysis. The developed method had a good repeatability and stability.

### Metabolic variation and biomarker identification

**Figure 3:**
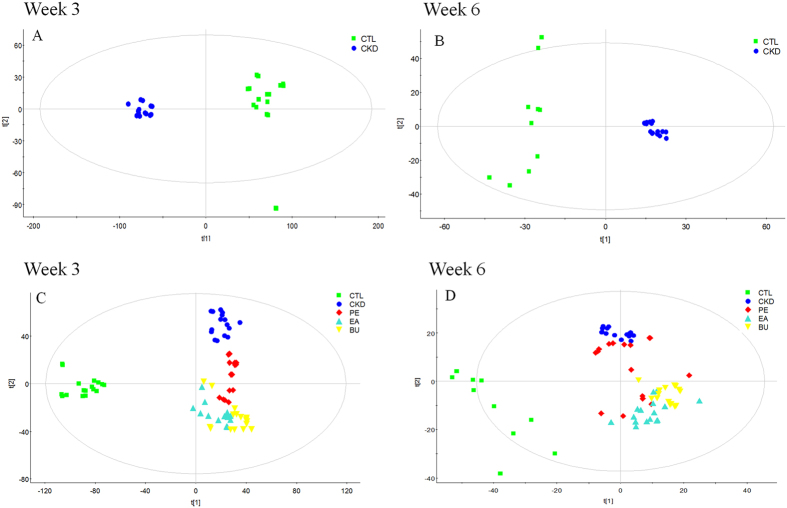
PCA scores plot of comparing control group and CKD group at week 3 (A) and week 6 (B); PCA scores plot of comparing control, CKD, CKD+PE, CKD+EA and CKD+BU groups at week 3 (C) and week 6 (D).

Metabolic profile of urine samples was acquired using UPLC-QTOF/HDMS in the positive ESI mode. The base peak intensity (BPI) chromatograms of urine samples from each group are shown in supplementary Figure S2. The PCA analyses were performed on the urine metabolite concentrations between the control and CKD group at week 3 (Fig. 3A) and week 6 (Fig. 3B) as well as among control, CKD, CKD+EA, CKD+PE and CKD+BU groups at week 3 (Fig. 3C) and week 6 (Fig. 3D). A clear separation in metabolic states was observed between the control and CKD groups at weeks 3 and 6 (Fig. 3A,B), pointing to marked alteration of urinary metabolites in this CKD model. On the other hand, CKD+EA and CKD+BU affected the metabolite variations in different directions and PCA analysis showed a clear separation between these two groups and the untreated CKD group (Fig. 3C,D). However, as shown in Fig. 3C,D, the impact of treatment in the CKD+PE group was less intense than that observed in the CKD+EA group, suggesting that PE extract is less potent than the other extracts.

**Figure 4:**
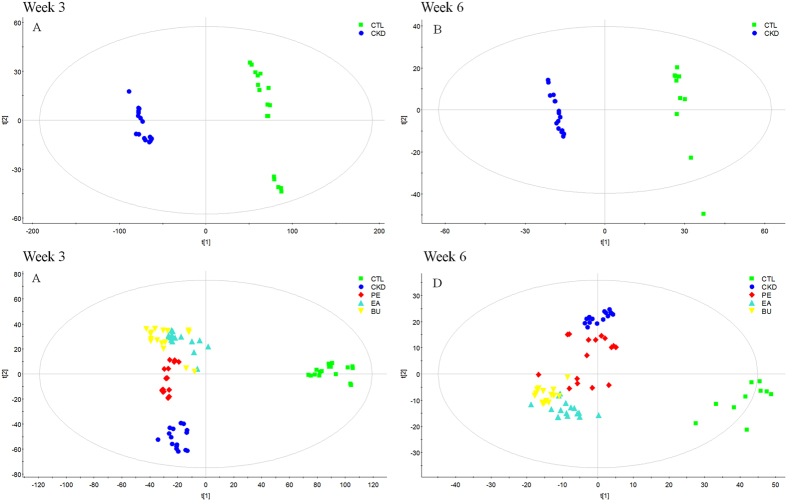
OPLS-DA score plots generated from the OPLS-DA of the QTOF/HDMS data from control group and CKD group at week 3 (**A**) and week 6 (**B**); PLS-DA score plots from control, CKD, CKD+PE, CKD+EA and CKD+BU groups at week 3 (**C**) and week 6 (D). (**A**) *R*^*2*^*X* = *0.515, R*^*2*^*Y* = *0.996, Q*^*2*^ = *0.993*; (**B**) *R*^*2*^*X* = *0.356, R*^*2*^*Y* = *0.996, Q*^*2*^ = *0.955*; and (**C**) *R*^*2*^*X* = *0.618, R*^*2*^*Y* = *0.983, Q*^*2*^ = *0.938*. (**D**) *R*^*2*^*X* = *0.416, R*^*2*^*Y* = *0.982, Q*^*2*^ = *0.840*.

**Figure 5:**
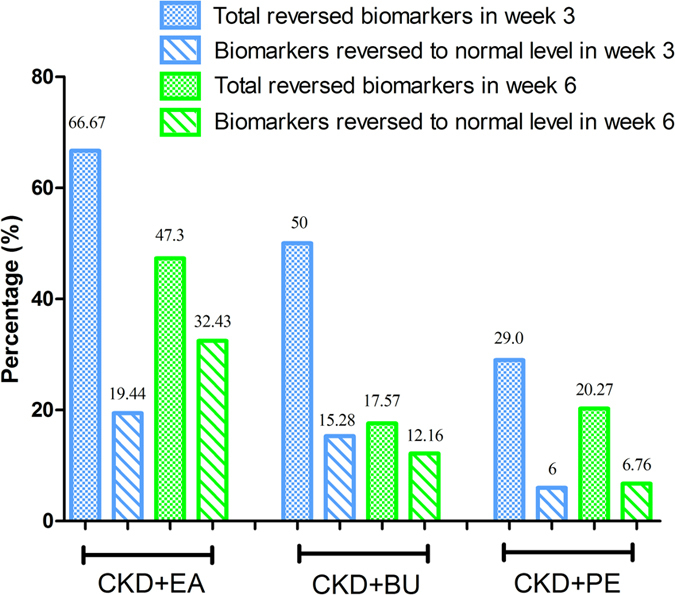
Percentage of the reversed biomarkers in the total biomarkers at week 3 and 6; Percentage of the biomarkers that reversed to normal level in the total biomarkers at weeks 3 and 6. This figure reflected the therapeutic effect of EA, BU and PE treatments on CKD rats.

Orthogonal partial least squares-discriminant analysis (OPLS-DA) was employed for identification of metabolite changes from control and CKD groups. The OPLS-DA score plot showed good fitness and high predictability of model with high statistical values of R^2^ and Q^2^ (Fig. 4). Differentially expressed metabolites were identified according to the previously reported method^15^. Seventy-three differentially expressed metabolites identified between the control and CKD groups at week 3 are listed in supplemental Table S1. The differences were determined by using the VIP values (>1.0) from OPLS-DA combined with the ANOVA (p < 0.05) with an FDR < 0.05 and Mann-Whitney U test (p < 0.05). In addition, we compared the concentration of these seventy-three metabolites among all groups including CKD animals treated with different extracts (Table S1). Using the above mentioned approach we identified seventy-four differentially expressed metabolites between the control and CKD groups at week 6 and compared the concentration of these seventy-four metabolites among the study groups (Table S2). Analysis of the data presented in Table S1 and Table S2, revealed that treatment with EA, BU and PE extracts exerted some therapeutic effects by normalizing or partially reversing the CKD-induced alterations in the biomarker metabolites (Fig. 5). This result is consistent with the observed improvements in serum biochemical parameters and histological and immunohistochemical findings. Furthermore, EA treatment showed better therapeutic effect than BU and PE treatments. Interestingly, percentage of the reversed biomarkers from EA treatment at week 6 is lower than that at week 3, nevertheless the percentage of biomarkers that reversed to normal level from EA treatment at week 6 is much higher than that seen at week 3. This result indicated that EA treatment was able to ameliorate the metabolism in CKD rat from 3 weeks to 6 weeks. But the therapeutic effect of BU and PE treatments decreased at week 6.

**Figure 6:**
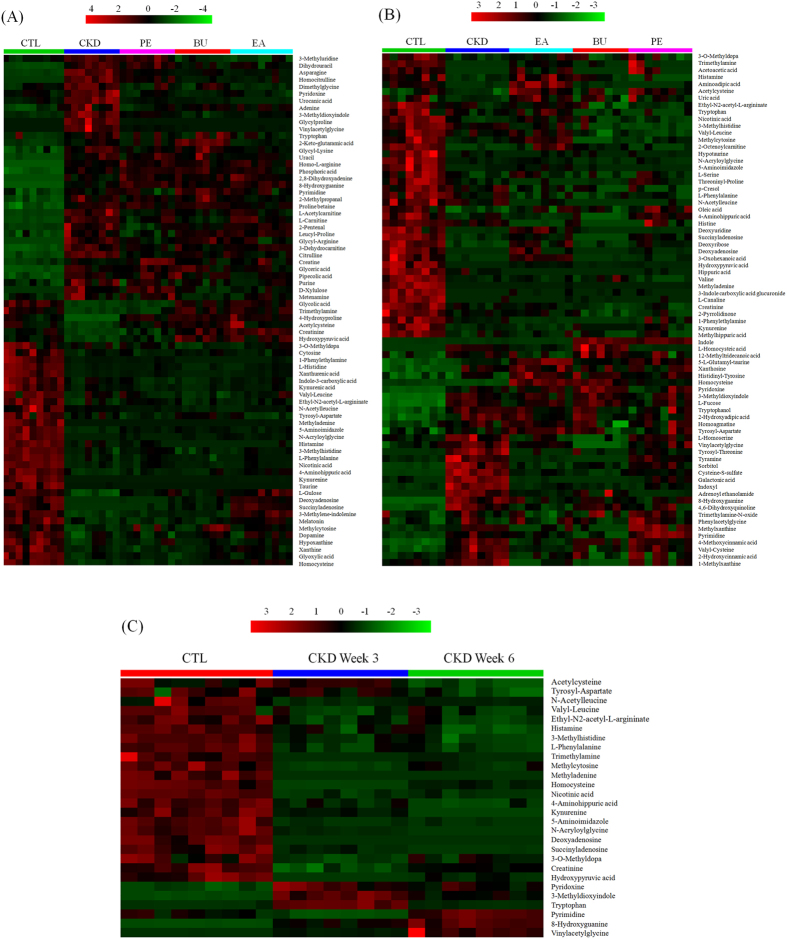
Heat maps for urinary metabolites at weeks 3 (A) and 6 (B) and the biomarkers appeared at both weeks 3 and 6 (C). The color of each section is proportional to the significance of change of metabolites (red, upregulated; green, downregulated). Rows: metabolites; Columns: samples.

**Figure 7:**
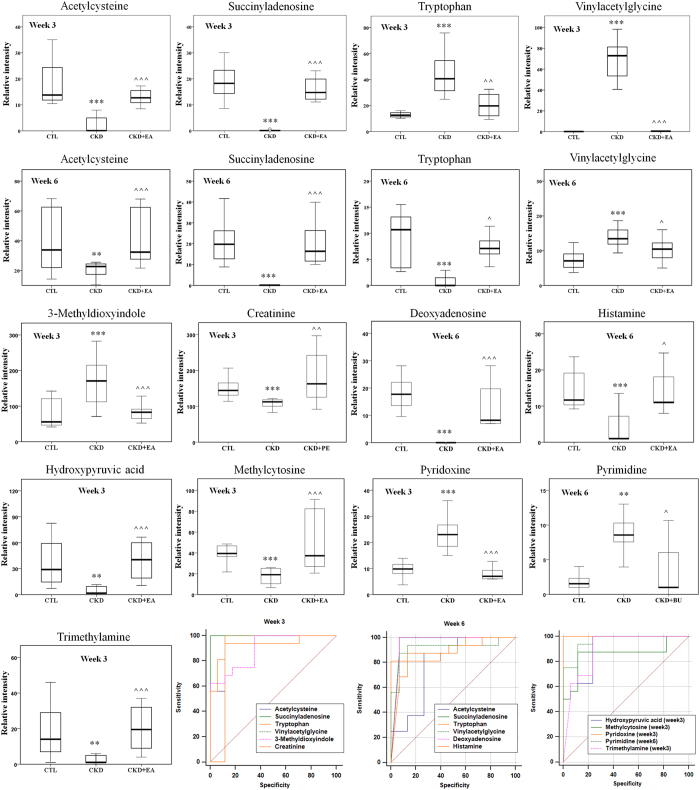
Therapeutic effect of EA, BU and PE extracts on CKD rats and receiver operating characteristic (ROC) curve of 28 biomarkers. Box plots showing relative abundance of 13 identified biomarkers that were completely reversed to normal level by treatment with EA, BU or PE extracts in week 3 or week 6 and their ROC curve. *p < 0.05, **p < 0.01, ***p < 0.001 significant difference compared to the control group; ^p < 0.05, ^^p < 0.01, ^^^p < 0.001 significant difference compared to the CKD group.

**Figure 8:**
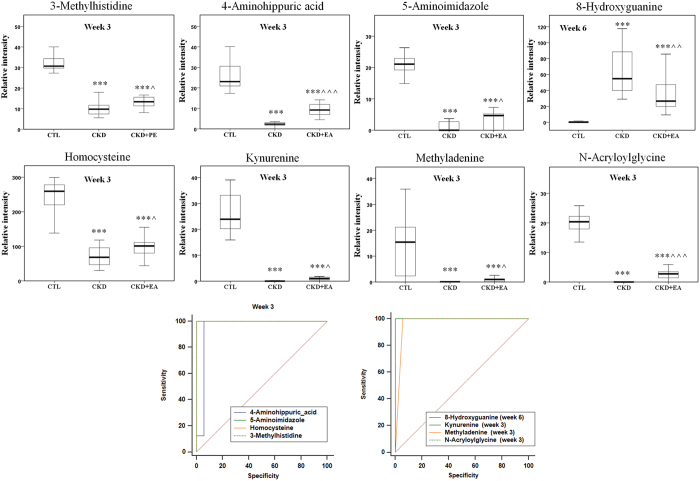
Therapeutic effect of EA, BU and PE extracts on CKD rats and receiver operating characteristic (ROC) curve of 28 biomarkers. Box plots showing relative abundance of 8 identified biomarkers that were reversed by treatment with EA, BU or PE extracts in week 3 or week 6 and their ROC curve. *p < 0.05, **p < 0.01, ***p < 0.001 significant difference compared to the control group; ^p < 0.05, ^^p < 0.01, ^^^p < 0.001 significant difference compared to the CKD group.

**Figure 9:**
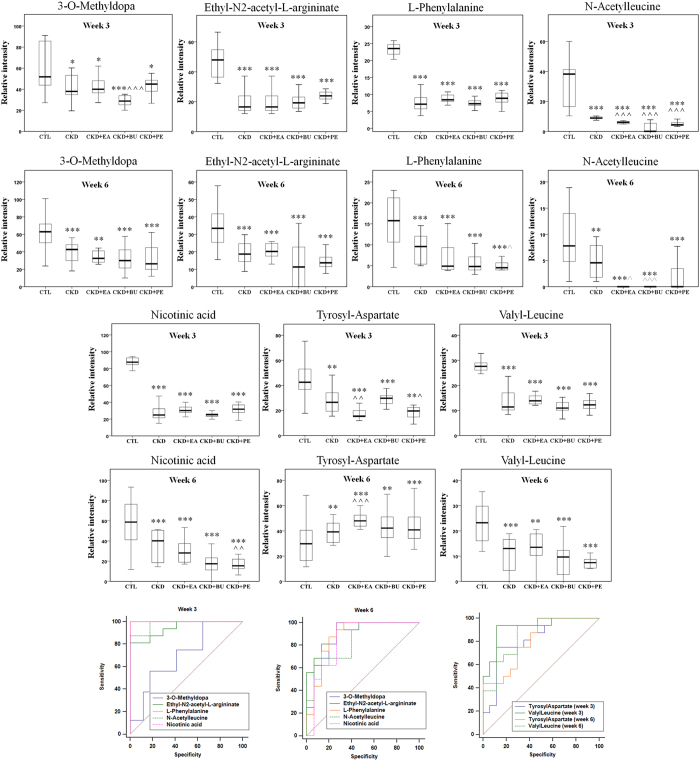
Therapeutic effect of EA, BU and PE extracts on CKD rats and receiver operating characteristic (ROC) curve of 28 biomarkers. Box plots showing relative abundance of 7 identified biomarkers that can’t be reversed by treatment with EA, BU or PE extracts in week 3 or week 6 and their ROC curve. *p < 0.05, **p < 0.01, ***p < 0.001 significant difference compared to the control group; ^p < 0.05, ^^p < 0.01, ^^^p < 0.001 significant difference compared to the CKD group.

It is worth mentioning that among the differential metabolites at weeks 3 and 6, twenty-eight differential metabolites were shared as being potentially characteristic of CKD (Table 1). To further understand the metabolic changes among different groups, the metabolites were visualized in a clustering heatmap which showed directly the variation of each metabolite. Figure 6A,B illustrates the identified differentially expressed metabolites in CKD groups that were up-regulated (red) or down-regulated (green) compared with control group at weeks 3 and 6, respectively. Figure 6C presented the heatmap built for 28 differential metabolites appearing both at weeks 3 and 6. As shown in Fig. 6, we found that this model was capable of distinguishing CKD group from control group and that EA, BU and PE treatments ameliorates CKD-induced metabolic abnormalities by influencing multiple metabolic pathways. Thirteen metabolites with the green color, including acetylcysteine, succinyladenosine, tryptophan, vinylacetylglycine among others, are listed in Table 1. Their abundances were completely restored to normal level with EA, BU or PE treatments within 3 or 6 week (Fig. 7). The abundance of eight metabolites (shown in dark color and listed in Table 1) was changed with EA, BU or PE treatments to the levels that are significantly different from those found in the untreated CKD group at week 3 or 6 (Fig. 8). The abundances of remaining eight blue metabolites did not improved with EA, BU or PE treatments at week 3 or 6 (Fig. 9).

## Discussion

**Figure 10:**
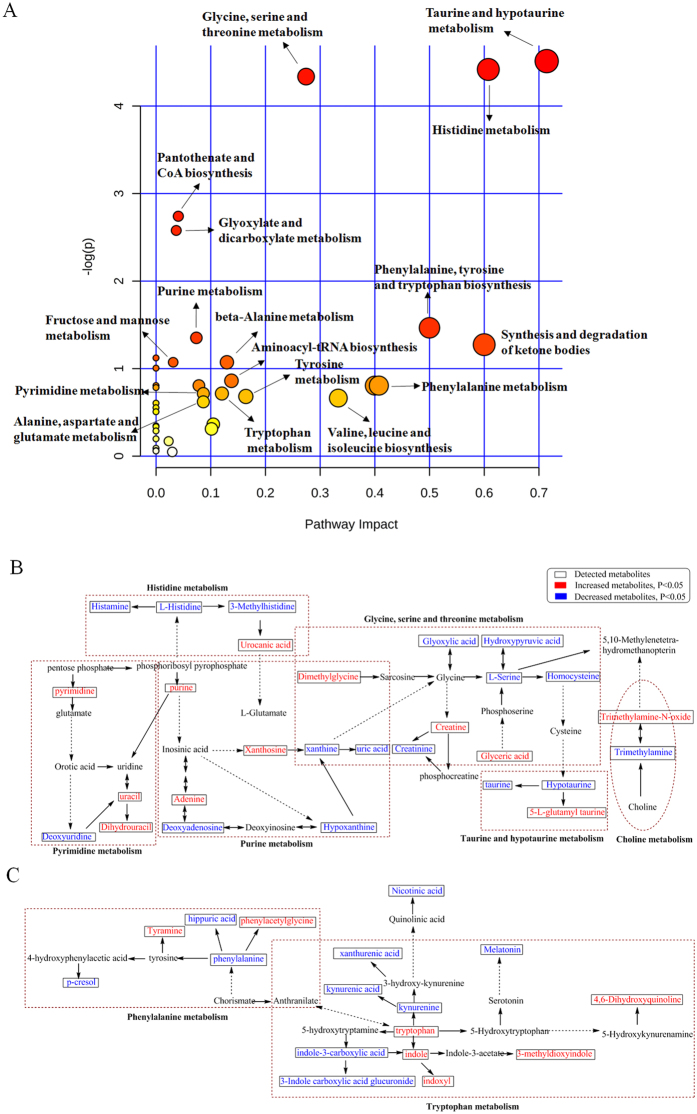
Pathway analysis of identified metabolites in the different groups. (**A**) On the basis of all differential metabolites (Table S1 and S2 in the Supporting Information), global metabolic disorders of the most relevant pathways induced by adenine were revealed using the MetaboAnalyst; small p value and big pathway impact factor indicate that the pathway is greatly influenced. (**B**) Histidine metabolism, glycine, serine and threonine metabolism, taurine and hypotaurine metabolism, purine metabolism, pyrimidine metabolism and choline metabolism. (**C**) Phenylalanine metabolism and tryptophan metabolism. Red color represents increased metabolites in CKD rats; Blue color represents decreased metabolites in CKD rats. Dotted arrow indicates multiple processes. Solid arrow indicates single process.

As shown by Yokozawa *et al.*^20^ rats fed adenine-containing diet developed chronic kidney disease marked by severe chronic tubulointerstitial nephropathy as confirmed by biochemical, histological and immunohistochemical examinations. UPLC-QTOF/HDMS-based urinary metabolomics revealed perturbations of the pathways of metabolism of amino acids (taurine and hypotaurine) as well as purine and pyrimidine in the CKD animals (Fig. 10). Treatment with the EA, BU and PE extracts of rhubarb ameliorated renal lesions, kidney function and urinary metabolite abnormalities in rats with adenine-induced CKD. In particular treatment with EA extract was more effective than BU and PE extracts in restoring the urinary metabolites toward normal levels.

### Changes in urinary amino acids and their metabolites

The identified metabolites including histamine, 3-methylhistidine, urocanic acid and L-histidine are byproducts of the histidine metabolism. Marked elevation of urocanic acid and reduction of histamine, 3-methylhistidine and L-histidine were observed in the CKD group, pointing to the disturbance in the pathway of histidine metabolsm (Fig. 10B). Histidine can be converted to histamine, 3-methylhistidine or urocanic acid via different pathways. In our study, urine histidine was significantly decreased in the CKD group, pointing possible reduction of its biosynthesis or its enhanced catabolism in the CKD animals. Low level of histidine may be responsible for the reduction of its downstream metabolites histamine and 3-methylhistidine. Histidine is an anti-inflammatory and anti-oxidant factor. These protective effects are attributed to the capacity of its imidazole ring to scavenge reactive oxygen species generated by cells during the inflammatory response^21^. In fact Watanabe *et al.* have reported the association of decreased plasma histidine with protein-energy wasting, systemic inflammation, oxidative stress and increased mortality in patients with CKD^22^.

Histamine, a product of histidine metabolism, plays an important role in the regulation of physiological and pathological functions. Several studies have reported increased plasma histamine in patients with CKD^23^,^24^, and its possible role in the pathogenesis of the associated glomerular and vascular endothelial damage and dysfunction^24^. The change of the urinary excretion of metabolites can reflect their altered rate of glomerular filtration, plasma concentration, intra-renal production/consumption, and or their handling by renal tubular epithelial cells. Urinary histamine level was significantly reduced in our untreated CKD rats. The underlying mechanism of the observed reduction of urinary histamine in the CKD group is presently unclear. However it may be in part due to the depletion of its precursors, histidine and 3-methylhistidine. Histidine and 3-methylhistidine are biomarkers of the rate of myofibrillar protein breakdown and their production is increased in patients with severe injury, thyrotoxicosis, neoplastic diseases and prednisolone therapy. Using ^1^H NMR approach Choi *et al.*^25^ found significant retention of multiple uremic toxins as well as myoinositol and 3-methylhistidine in CKD patients maintained on hemodialysis. Thus reduced urinary excretion found in our CKD rats may account for the reported retention of these compounds in CKD patients. Administration of EA extract of rhubarb restored urinary histamine and urocanic acid excretion to normal levels in our CKD rats. Treatment with PE extract prevented the decline in urinary L-histidine and 3-methylhistidine in the CKD rats. These findings demonstrate the efficacy of different rhubarb extracts in reversing the CKD-associated disturbance of histidine metabolism.

The untreated CKD group showed significant increase in urinary tyramine and phenylacetylglycine and significant reduction in urinary phenylalanine, hippuric acid and p-cresol excretion (Fig. 10C). Phenylalanine is an essential amino acid and the precursor of the catecholamines (epinephrine, tyramine, dopamine and norepinephrine) which are major neurotransmitters. Earlier studies have revealed urinary losses of phenylalanine in patients with CKD^26^ and reduction of urine phenylalanine concentration in CKD rats^19^. The observed reduction of urinary phenylalanine may be due to its depletion in our CKD rats. p-Cresol is an end-product of conversion of phenylalanine by intestinal bacteria which is sulfated by the liver and readily excreted in the urine of normal humans and animals as p-cresol sulfate. Serum p-cresol sulfate levels rises with progression of renal failure in patients and animals with CKD^27^. This is due to a combination of reduced renal excretion and increased production of p-Cresol by intestinal microbial flora^28^. As expected urinary p-Cresol level was significantly decreased in our CKD group, indicating reduced renal clearance of this gut derived uremic toxin. Phenylacetylglycine is another metabolite of phenylalanine whose metabolism is influenced by gut flora^29^,^30^. Earlier study has demonstrated profound changes in the structure and function of the gut microbial flora in humans and animals with CKD^31^. Given the impact of the gut microbial flora on metabolism of the amino acids, the observed changes in the above metabolites in our CKD rats can be, in part, due to the CKD-induced changes in the gut microflora. Treatment of CKD rats with the EA extract of rhubarb restored urine tyramine and phenylacetylglycine levels to values found in the normal control rats. However, treatment with the three rhubarb extracts failed to prevent CKD-induced changes in urine phenylalanine, hippuric acid or p-cresol.

The CKD group showed marked elevation of urine tryptophan, indole, indoxyl and 3-methyldioxyindole and significant reduction of urine kynurenine, kynurenic acid, xanthurenic acid, melatonin, indole-3-carboxylic acid and 3-indole carboxylic acid glucuronide indicating disturbance of tryptophan metabolic pathway (Fig. 10C). Tryptophan is an essential amino acid which participates in kynurenine and serotonin metabolic pathways. Increased urinary tryptophan found in our CKD rats is consistent with the results of the previously published studies^17^,^30^. Kynurenic acid is a metabolite of tryptophan which can be converted into kynurenine by indoleamine-2,3-dioxygenase. Kynurenine is then converted to kynurenic acid by aminotransferase. Serum kynurenic acid level has been shown to correlate with the level of soluble endothelial adhesion molecules and oxidative status in patients with CKD^32^. Zhao *et al.* have shown plasma kynurenic acid/tryptophan ratio to be a sensitive and reliable indicator of renal function that could serve as a biomarker to assess the risk or presence of kidney disease^33^. Mutsaers *et al.* have shown accumulation of indole-3-acetic acid, indoxyl sulfate and kynurenic acid in patients with CRF^34^. This phenomenon is, in part, due to reduced urinary excretion of these metabolites as shown in our previous study in CKD rats^19^. Byrd *et al.* reported disturbance of the indolic pathway of tryptophan metabolism in uraemia^35^. Taken together, these findings illustrate the disturbance of tryptophan metabolic pathways in CKD. Treatment with the EA extract of rhubarb restored the urine tryptophan, indoxyl and 3-methyldioxyindole levels to normal in CKD rats. Increased 4,6-dihydroxyquinoline and indole as well as decreased kynurenine, melatonin, indole, xanthurenic acid and 3-indole carboxylic acid glucuronide were reversed by EA or PE extracts. However neither EA, nor BU or PE extracts could prevent the changes of urine kynurenic acid and indole-3-carboxylic acid levels. These findings suggest that rhubarb extracts can improve most but not all CKD-associated alterations of tryptophan metabolism.

The CKD rats exhibited significant elevation of urine creatine, dimethylglycine and glyceric acid as well as significant reduction of creatinine, L-serine, homocysteine, hydroxypyruvic acid and glyoxylic acid suggesting disturbance of glycine, serine and threonine metabolism (Fig. 10B). Creatine is a nitrogenous organic acid which is essential for delivery of energy to skeletal muscle via formation of ATP followed by its conversion to creatinine. Creatinine is excreted in the urine and measurement of its serum concentration is the most commonly used indicator of renal function. The CKD rats employed in the present study exhibited significant rise in serum creatinine reflecting their reduced renal function. Total body creatinine production is directly related to the body muscle mass. CKD frequently results in muscle atrophy which can account for the decreased urinary creatinine in our CKD rats. Homocysteine is a well-established biomarker of renal dysfunction. Plasma homocysteine is increased significantly in patients with moderate renal failure and is markedly elevated in patients with end-stage renal disease (ESRD)^36^. Homocysteine metabolism is linked to betaine-homocysteine methyl transferase which catalyzes transfer of a methyl group from glycine betaine to homocysteine, producing methionine and dimethylglycine^37^. Dimethylglycine is a well-known inhibitor of betaine-homocysteine methyl transferase^38^. Dimethylglycine accumulates in uremia and contributes to elevated plasma homocysteine concentration in patients and animals with renal failure^37^. In addition reduced glomerular filtration of homocysteine contributes to its rise in body fluids and reduced urinary excretion. Treatment with EA and BU extracts of rhubarb reversed the rise in urine dimethylglycine, and the decline in urine creatinine and hydroxypyruvic acid to normal level in CKD rats. The reduction in urine homocysteine and glyoxylic acid was reversed by EA extract. However neither EA nor BU or PE extracts could prevent the changes in urine creatine, L-serine or glyceric acid. These observations suggest that rhubarb extracts can ameliorate some but not all CKD-associated abnormalities of glycine, serine and threonine metabolism.

### Changes in urinary purine metabolites

The CKD group showed markedly elevated urine adenine and xanthosine as well as significantly decreased urine deoxyadenosine, xanthine, hypoxanthine and uric acid suggesting disturbance of purine metabolism (Fig. 10B). Adenine is a purine base which when present in excess quantities, it is converted to 8-hydroxyadenine and eventually to 2,8-dihydroxyadenine by xanthine dehydrogenase^39^. Adenine and 2,8-dihydroxyadenine are excreted in the urine. However, due to its very low solubility, 2,8-dihydroxyadenine can precipitate in the renal tubules^40^. Uric acid, xanthine and hypoxanthine are oxidation byproducts of purine metabolism. Purine metabolic pathway includes transformation of hypoxanthine to xanthine and xanthine to uric acid by xanthine oxidase^41^,^42^. Plasma concentrations of hypoxanthine and uric acid are increased in patients with ESRD and significantly fall after hemodialysis; however, their mean concentrations are generally higher than normal values^43^. High concentration of uric acid in blood (hyperuricemia) can lead to deposition of urate crystals causing arthritis, renal interstitial fibrosis and kidney stone formation^44^. In fact lowering uric acid level has been shown to slow CKD progression^45^ and attenuate tubulointerstitial fibrosis in rats with CKD induced by 5/6 nephrectomy^46^ and diabetic nephropathy^47^. Treatment with EA extract reversed the elevation of urine adenine and the reductions of urine deoxyadenosine, and uric acid to normal level in CKD rats. In addition the reduction in urine xanthine was reversed by EA extract. However neither EA nor BU or PE extracts could prevent the CKD-induced changes in urine hypoxanthine or xanthosine levels. Thus rhubarb extracts partially ameliorated the CKD-associated abnormalities of purine metabolism.

### Changes of urine taurine and choline metabolites

Our CKD rats had significantly elevated urine 5-L-glutamyl-taurine and significantly decreased taurine and hypotaurine, indicating disturbance of taurine metabolism (Fig. 10B). Taurine has numerous vital functions. It facilitates transport of various ions such as sodium, potassium, calcium, and magnesium, exerts cardiotonic actions, participates in osmoregulation, stabilizes membrane potential in skeletal muscle, has anti-oxidant and anti-inflammatory properties, and acts as a neurotransmitter^48^. Patients with ESRD have been shown to be taurine depleted with low plasma and muscle tissue concentrations of taurine^49^. In addition urinary taurine is significantly reduced in patients with advance CKD^26^. The reduction in urine taurine found in our CKD rats is consistent with the reported findings in patients with advance CKD. Hypotaurine is an intermediate in the biosynthesis of taurine and possesses anti-oxidant activity^50^. In an earlier study Hayashi *et al.* found decreased urine hypotaurine but elevated serum hypotaurine and taurine in patients with CKD^51^. Urine hypotaurine was markedly decreased in our CKD rats confirming the results of the latter study in humans with CKD.

The CKD rats employed in our study exhibited significantly elevated urine trimethylamine-N-oxide (TMAO) and decreased trimethylamine (TMA) (Fig. 10B). TMA is a byproduct of decomposition of choline by gut microbial flora. TMAO which is a toxic metabolite is the product of TMA oxidation. TMA and TMAO accumulate in the body fluids between hemodialysis sessions in patients with ESRD and are efficiently removed by hemodialysis^52^. Hauet *et al.* demonstrated that TMAO correlated with the degree of renal injury inflicted by different mechanisms^53^,^54^. The bacteria in the human gut convert dietary carnitine and dietary lecithin to TMAO^55^, Treatment with the EA extract reversed the increase in TMAO and the decline in TMA to normal levels in CKD rats, demonstrating the efficacy of rhubarb extract in ameliorating the changes in urinary choline metabolites. However none of the three rhubarb extracts could prevent the changes in urine taurine metabolites.

## Conclusions

CKD rats with adenine-induced chronic interstitial nephropathy exhibited marked abnormalities in urinary amino acids, purine and taurine metabolites. Administration of the EA, BU or PE extracts of rhubarb attenuated the extent of kidney injury and dysfunction and either fully or partially reversed the changes in the urinary metabolites in the treated CKD animals. Further studies are needed to explore the underlying mechanism(s) of CKD-induced abnormalities of the identified urinary metabolites and the salutary actions of the rhubarb extracts.

## Materials and Methods

### Chemicals and reagents

Creatinine, L-tryptophan, valine, and p-cresol sulfate were obtained from the National Institutes for Food and Drug Control and Amresco Company. Transforming growth factor beta 1 (TGF-β1) antibody was purchased from Santa Cruz Biotechnology. LC-grade methanol and acetonitrile were purchased from the Baker Company. Ultra high purity water was prepared using a Milli-Q water purification system. Other chemicals were of analytical grade and their purity was above 99.5%.

### Preparation of Rhubarb Extracts

Rhubarb was ground to powder (about 20 meshes) by a disintegrator. Rhubarb powder (2 kg) was weighed and extracted with 15 L 95% ethanol for 0.5 h by ultrasonic method for three times. The collected extract was concentrated under pressure to yield a brown ethanol extract (Rub). Then the ethanol extract obtained was partitioned between water and organic solvents of increasing polarities, to yield three new fractions including petroleum ether extract (PE), ethyl acetate extract (EA) and n-butanol extract (BU).

### Animals and Sample Collection

Male Sprague-Dawley rats, weighting 200 ± 10 g were purchased from Fourth Military Medical University (Xi’an, China). The rats were randomized to divide into the following five groups (n = 8): control group, CKD group, CKD+EA group, CKD+BU group and CKD+PE group. CKD, CKD+EA, CKD+BU and CKD+PE groups were then given 200 mg/kg body weight of adenine dissolved in 1% (w/v) gum acacia solution by oral gavage once everyday continuously for three weeks^56^,^57^. Control group was similarly provided an equal volume of gum acacia solution. During the adenine gastric gavage periods, after 3 h, CKD+EA, CKD+BU and CKD+PE groups were administered EA extract (20 mg/mL), BU extract (60 mg/mL) and PE extract (80 mg/mL) by gastric irrigation respectively during six weeks study periods. All groups were only administered by oral gavage with the 1% (w/v) gum acacia solution. Before urine collection, CKD+EA, CKD+PE and CKD+BU group rats stop being administrated EA, BU and PE extract for two days to reduce disturbance of chemical components of rhubarb. The individual rats were placed in metabolic cages (1 rat/cage) to obtain 24 h urine in the 3th and 6th week. All the samples were stored at −80 °C before analysis. This study was approved by the Ethical Committee of Northwest University, and all procedures were in accordance with the Helsinki Declaration.

### Biochemical determination

Plasma and urine biochemistry was analyzed as described in detail previously^58^.

### H&E staining and Masson’s trichrome staining

All the kidney tissues were excised, fixed in 4% formaldehyde and embedded in paraffin. Kidney tissue sections (5 μm) were obtained and used for histological and immunohistochemical analyses. H&E staining was performed according to the standard H&E protocol. The injury score was acquired by modified Banff classification criteria in ten randomly selected non-overlapping fields per rat H&E stained kidney tissues^59^. All specimens were blindly evaluated by one nephrologist.

For renal fibrosis, Masson’s trichrome staining from the each group was used to evaluate the extent of renal fibrosis according to the standard Masson’s trichrome protocol. Briefly, kidney tissue sections were successively immersed into Weigert’s iron hematoxylin, Biebrich scarlet-acid fuchsin, phosphomolybdic-phosphotungstic acid, and aniline blue. To quantify the renal fibrosis, the blue pixel contents of the images were photographed with the same microscope and magnification times. Ten different views in each group were selected to detect the values of the integral optical density and the total area and the expression intensity was calculated as the percentage of the integral optical density to the total area which was performed by Image-Pro Plus 6.0 (Media Cybernetics, Inc.).

### Polarized microscopy of picrosirius red stained kidney tissue collagen

Picrosirius red staining was one of the best staining techniques of collagen deposition. The birefringence was highly specific for collagen expression. In bright-field microscopy, collagen was red on a pale yellow background. When observed using polarized light microscopy, the larger collagen fibers were bright yellow or orange and the thinner ones (type I collagen) and reticular fibers were green (type III collagen). The expression of types I and III collagen was examined by Picrosirius red staining and polarization method^60^. Briefly, kidney tissue sections stained with a freshly prepared 0.1% of Sirius red in saturated aqueous picric acid. After rinsing twice in 0.01 mol/L hydrochloric acid and distilled water, sections were dehydrated and mounted in Permount. Ten polarized micrographs in each group were randomly selected for observation under polarized light microscopy and images of the same areas were taken under the same exposure time before and after 90° stage rotation and compared. Image analysis was done by using above-mentioned Image-Pro Plus 6.0 software and semi-quantitative methods.

### Immunohistochemical staining

TGF-β1 immunohistochemical staining was performed as described in detail previously^61^. Briefly, kidney tissue sections were deparaffinised in xylene, hydrated using graded ethanol, and rinsed with tap water and distilled water. Then the endogenous peroxide activity was blocked using 0.3% hydrogen peroxide in methanol for 30 min. For antigen retrieval, the kidney tissue sections were incubated with 10 μmol/L citrate buffer solution (pH: 6.0) and boiled for 10–15 min. Subsequently, the kidney tissue sections were blocked with 10% normal goat serum for 1 h at room temperature and then incubated overnight at 4 °C with rabbit primary antibody TGF-β1 (Santa Cruz Biotechnology, CA, USA). After washing with PBS, the secondary antibody was added and the sections were incubated at 37 °C for 1 h. Finally, the kidney tissue sections were exposed to diaminobenzidine peroxidase substrate for 5 min and counterstained with hematoxylin and eosin. Image analysis was done by using above-mentioned Image-Pro Plus 6.0 software and semi-quantitative methods.

### Urine preparation and UPLC-MS analysis

Urine samples were prepared as described previously^19^. Metabolomics was performed on a Waters Acquity^TM^ Ultra Performance LC system equipped with a Waters Xevo^TM^ G2-S QTof MS. chromatographic separation, mass spectrometry were described in detail in the Supplementary information.

### Data processing and statistical analysis

The precision and reproducibility of this experiment were tested for assessment of the developed UPLC-MS method following the reported method^57^. The mass data acquired were imported to Markerlynx XS and Progenesis QI for peak detection and alignment. The resulting three-dimensional matrix, including arbitrary compound index (paired retention time-m/z), sample names (observations), and normalized peak areas (variables) were introduced into the SIMCA-P 13.0 Software package (Umetrics, Umeå, Sweden) for multivariate statistical analysis. The auto-scaled, normalized spectral data were conducted by principal component analysis (PCA) to visualize general clustering, trends, or outliers among the observations. Orthogonal Partial least-square-discriminant analysis (OPLS-DA) was utilized to validate the PCA model and identify the differential metabolites accountable for adenine-induced CKD.

Fold changes (each group/control group or each group/CKD group) of adenine-induced CKD and the area under the curve (AUC) were calculated by Metaboanalysis 3.0. Classical one-way analysis of variance (ANOVA) and Mann-Whitney test were used to judge the statistical significance of the results by SPSS 19.0. Variable importance in the projection (VIP) ranks the overall contribution of each variable to the OPLS-DA model, and those variables with VIP >1.0 are considered relevant for group discrimination^62^. The critical p-value of both tests was set to 0.05 for this study. False discovery rate (FDR)^63^,^64^, a statistical approach to the problem of multiple comparisons, was used in this study to verify the discriminant metabolites chosen by ANOVA-p values (<0.05).

## Additional Information

**How to cite this article**: Zhang, Z.-H. *et al.* Metabolomics insights into chronic kidney disease and modulatory effect of rhubarb against tubulointerstitial fibrosis. *Sci. Rep.* **5**, 14472; doi: 10.1038/srep14472 (2015).

## Supplementary Material

Supplementary Information

## Figures and Tables

**Table 1:** Differential metabolites from different groups at weeks 3 and 6.

Metabolites	CRF VS Control week 3								CRF VS Control week 6							
	VIP	*p*^a^	*p*^b^	FDR^c^	FC^d^	AUC^e^	Sensitivity^f^	Specificity^f^	VIP	*p*^a^	*p*^b^	FDR^c^	FC^d^	AUC^e^	Sensitivity^f^	Specificity^f^
3-Methyldioxyindole	6.11	3.52E-05	1.16E-04	4.22E-05	1.04	0.89	100.0	64.7	12.53	1.90E-09	3.10E-06	1.78E-08	1.78	0.99	93.8	100.0
3-Methylhistidine	3.73	3.84E-16	9.63E-07	3.11E-15	−1.99	1.00	100.0	100.0	2.69	4.48E-05	2.33E-04	8.01E-05	−1.87	0.89	100.0	73.3
3-O-Methyldopa	2.33	1.54E-02	5.18E-02	1.54E-02	−0.32	0.70	56.3	82.4	3.79	2.54E-04	3.22E-04	3.68E-04	−1.36	0.88	93.8	73.3
4-Aminohippuric acid	3.38	1.69E-10	1.11E-05	3.62E-10	−3.65	0.95	100.0	94.1	2.71	3.70E-05	3.22E-04	6.91E-05	−0.71	0.88	62.5	100.0
5-Aminoimidazole	3.64	3.48E-12	7.47E-07	9.41E-12	−5.50	1.00	100.0	100.0	3.38	1.67E-08	2.83E-06	8.26E-08	−5.97	0.98	93.8	100.0
8-Hydroxyguanine	8.79	1.56E-11	7.47E-07	3.92E-11	8.76	1.00	100.0	100.0	7.34	1.11E-09	1.72E-06	1.17E-08	7.53	1.00	100.0	100.0
Acetylcysteine	2.82	6.36E-06	9.02E-06	8.29E-06	−4.54	0.95	100.0	88.2	3.44	2.86E-03	7.19E-03	3.48E-03	−0.42	0.78	87.5	73.3
Creatinine	2.87	3.59E-05	2.93E-05	4.23E-05	0.43	0.93	93.8	88.2	7.08	6.79E-05	3.22E-04	1.16E-04	−1.16	0.88	87.5	80.0
Deoxyadenosine	2.96	1.61E-12	3.46E-07	4.71E-12	−7.18	1.00	100.0	100.0	3.33	1.01E-07	4.58E-06	3.40E-07	−6.46	0.96	100.0	93.3
Ethyl-N2-acetyl-L-argininate	3.59	2.36E-08	1.31E-05	3.51E-08	−1.19	0.94	81.3	100.0	3.28	5.34E-06	4.67E-05	1.28E-05	−0.87	0.93	100.0	73.3
Histamine	2.10	4.40E-13	9.24E-07	1.40E-12	−3.29	1.00	100.0	100.0	2.47	3.61E-04	3.20E-04	4.97E-04	−4.20	0.87	87.5	86.7
Homocysteine	10.26	4.12E-13	9.63E-07	1.37E-12	−1.74	1.00	100.0	100.0	2.10	5.54E-08	4.19E-06	2.11E-07	−3.30	0.98	62.5	66.7
Hydroxypyruvic acid	4.27	2.24E-03	8.61E-05	2.27E-03	−3.09	0.90	100.0	76.5	5.08	2.62E-03	1.03E-06	3.23E-03	−7.13	1.00	100.0	100.0
Kynurenine	3.94	9.78E-15	5.16E-07	5.49E-14	−7.71	1.00	100.0	100.0	4.04	4.73E-07	2.86E-06	1.42E-06	−4.56	0.99	100.0	93.3
L-Phenylalanine	3.17	1.71E-18	9.63E-07	3.13E-17	−1.64	1.00	100.0	100.0	2.23	4.03E-05	1.39E-04	7.37E-05	−2.12	0.88	87.5	80.0
Methyladenine	2.66	1.01E-04	7.00E-07	1.15E-04	−6.03	0.97	100.0	94.1	2.98	3.98E-08	4.59E-06	1.59E-07	−6.38	0.93	100.0	86.7
Methylcytosine	2.92	1.49E-04	4.76E-04	1.68E-04	−1.10	0.86	87.5	88.2	2.52	3.61E-03	8.08E-03	4.16E-03	−1.85	0.78	43.8	100.0
N-Acetylleucine	3.47	1.92E-05	2.83E-06	2.42E-05	−1.66	0.98	87.5	100.0	1.97	2.72E-04	1.34E-03	3.87E-04	−2.17	0.84	62.5	93.3
N-Acryloylglycine	3.69	4.63E-11	1.96E-07	1.06E-10	−7.65	1.00	100.0	100.0	3.69	2.01E-08	6.13E-06	8.90E-08	−4.96	0.97	87.5	100.0
Nicotinic acid	6.29	2.33E-19	9.63E-07	1.70E-17	−1.65	1.00	100.0	100.0	4.00	1.99E-04	8.99E-04	3.03E-04	−0.83	0.85	100.0	73.3
Pyridoxine	2.74	2.16E-09	9.63E-07	3.93E-09	1.30	1.00	100.0	100.0	4.30	3.71E-09	2.55E-06	2.59E-08	2.18	1.00	93.8	100.0
Pyrimidine	3.24	2.74E-08	5.66E-06	4.01E-08	0.86	0.96	93.8	88.2	1.90	3.77E-04	6.51E-03	5.10E-04	1.59	0.78	75.0	93.3
Succinyladenosine	3.21	1.99E-12	5.16E-07	5.58E-12	−7.25	1.00	100.0	100.0	3.69	6.50E-08	3.18E-06	2.27E-07	−6.89	0.96	100.0	93.3
Trimethylamine	2.98	1.41E-03	8.50E-05	1.49E-03	−7.70	0.90	100.0	76.5	3.15	3.39E-03	1.53E-05	3.96E-03	−6.03	0.90	100.0	80.0
Tryptophan	4.25	8.91E-04	1.79E-04	9.71E-04	1.48	0.88	100.0	88.2	2.00	1.63E-04	7.41E-05	2.63E-04	−4.39	0.91	81.3	100.0
Tyrosyl-Aspartate	2.61	6.50E-04	1.72E-03	7.18E-04	−0.61	0.82	75.0	88.2	2.46	3.45E-04	1.79E-03	4.84E-04	0.65	0.83	100.0	60.0
Valyl-Leucine	2.82	1.13E-06	4.02E-05	1.56E-06	−0.88	0.92	93.8	88.2	2.61	3.55E-05	2.75E-04	6.77E-05	−2.22	0.88	93.8	73.3
Vinylacetylglycine	6.46	3.60E-08	2.67E-07	5.15E-08	9.08	1.00	100.0	100.0	2.04	2.73E-05	7.72E-05	5.33E-05	1.41	0.92	87.5	93.3

^a^Variable importance in the projection (VIP) was obtained from the PLS-DA model.

^b^The p value was calculated from ANOVA.

^c^The p value was calculated from nonparametric test Mann−Whitney U test.

^d^Fold change was calculated as a binary logarithm of the average mass response (normalized peak area) ratio between each group vs control group or between each group vs CKD group, where a positive value means that the average mass response of the metabolite in each group is larger than that in the control group.

^e^Area under the receiver operating characteristic (ROC) curve, with the 95% confidence interval (CI) range in parentheses.

^f^Sensitivity and Specificity were calculated from ROC curve and their unit is percent. Metabolites with green color indicate that their concentration were reversed to normal level with EA, BU or PE treatments; Metabolites with dark color indicate that their concentration were reversed with EA, BU or PE treatments; Metabolites with blue color indicate that their concentration can’t be reversed with EA, BU and PE treatments.

## References

[b1] Lameire N., Jager K., Van Biesen W., de Bacquer D. & Vanholder R. Chronic kidney disease: a European perspective. Kidney Int. 68, S30–38 (2005). 10.1111/j.1523-1755.2005.09907.x 16336574

[b2] Levey A. S. *et al.* Chronic kidney disease as a global public health problem: approaches and initiatives - a position statement from Kidney Disease Improving Global Outcomes. Kidney Int. 72, 247–59 (2007). 17568785 10.1038/sj.ki.5002343

[b3] Zeisberg M. & Neilson E. G. Mechanisms of tubulointerstitial fibrosis. J. Am. Soc. Nephrol. 21, 1819–34 (2010). 20864689 10.1681/ASN.2010080793

[b4] Agarwal S. K., Singh S. S., Lakshmi V., Verma S. & Kumar S. Chemistry and pharmacology of rhubarb (Rheum species)—a review. J. Sci. Ind. Res. 60, 1–9 (2001).

[b5] Kashiwada Y., Nonaka G. I. & Nishioka I. Studies on Rhubarb (Rhei Rhizoma). XV: Simultaneous determination of phenolic constituents by high-performance liquid chromatography. Cheml. Pharm. Bull. 37, 999–1004 (1989).

[b6] Wang J. *et al.* Assessment of the renal protection and hepatotoxicity of rhubarb extract in rats. J. Ethnopharmacol. 124, 18–25 (2009). 19376216 10.1016/j.jep.2009.04.018

[b7] Mitsuma T., Yokozawa T., Oura H. & Terasawa K. [Rhubarb therapy in patients with chronic renal failure (Part 2)]. Nihon Jinzo Gakkai Shi 29, 195–207 (1987). 3599544

[b8] Li X. & Wang H. Chinese herbal medicine in the treatment of chronic kidney disease. Adv. Chronic Kidney Dis. 12, 276–81 (2005). 16010642 10.1016/j.ackd.2005.03.007

[b9] Yokozawa T., Suzuki N., Zheng P. D., Oura H. & Nishioka I. Effect of orally administered rhubarb extract in rats with chronic renal failure. Chem. Pharm. Bull. 32, 4506–13 (1984). 6532552 10.1248/cpb.32.4506

[b10] Zhao Y. Y. & Lint R. C. Metabolomics in nephrotoxicity. Adv. Clin. Chem. 65, 69–89 (2014). 25233611

[b11] Zhang A. *et al.* Metabolomics: towards understanding traditional Chinese medicine. Planta Med. 76, 2026–35 (2010). 21058239 10.1055/s-0030-1250542

[b12] Zhao Y. Y. Metabolomics in chronic kidney disease. Clin. Chim. Acta 422, 59–69 (2013). 23570820 10.1016/j.cca.2013.03.033

[b13] Zhao Y. Y. & Lin R. C. UPLC-MS^E^ application in disease biomarker discovery: the discoveries in proteomics to metabolomics. Chem. Biol. Interact. 215, 7–16 (2014). 24631021 10.1016/j.cbi.2014.02.014

[b14] Zhao Y. Y., Cheng X. L., Vaziri N. D., Liu S. & Lin R. C. UPLC-based metabonomic applications for discovering biomarkers of diseases in clinical chemistry. Clin. Biochem. 47, 16–26 (2014). 25087975 10.1016/j.clinbiochem.2014.07.019

[b15] Zhao Y. Y., Cheng X. L., Wei F., Bai X. & Lin R. C. Application of faecal metabonomics on an experimental model of tubulointerstitial fibrosis by ultra performance liquid chromatography/high-sensitivity mass spectrometry with MS^E^ data collection technique. Biomarkers 17, 721–729 (2012). 23020077 10.3109/1354750X.2012.724450

[b16] Zhao Y. Y. *et al.* Serum metabonomics study of adenine-induced chronic renal failure rat by ultra performance liquid chromatography coupled with quadrupole time-of-flight mass spectrometry. Biomarkers 17, 48–55 (2012). 22133066 10.3109/1354750X.2011.637180

[b17] Zhao Y. Y., Liu J., Cheng X. L., Bai X. & Lin R. C. Urinary metabonomics study on biochemical changes in an experimental model of chronic renal failure by adenine based on UPLC Q-TOF/MS. Clin. Chim. Acta 413, 642–649 (2012). 22227165 10.1016/j.cca.2011.12.014

[b18] Zhao Y. Y. *et al.* UPLC-Q-TOF/HSMS/MS^E^-based metabonomics for adenine-induced changes in metabolic profiles of rat faeces and intervention effects of ergosta-4,6,8(14),22-tetraen-3-one. Chem. Biol. Interact. 301, 31–38 (2013). 23246428 10.1016/j.cbi.2012.12.002

[b19] Zhao Y. Y., Li H. T., Feng Y. L., Bai X. & Lin R. C. Urinary metabonomic study of the surface layer of Poria cocos as an effective treatment for chronic renal injury in rats. J. Ethnopharmacol. 148, 403–10 (2013). 23612421 10.1016/j.jep.2013.04.018

[b20] Yokozawa T. *et al.* Animal model of adenine-induced chronic renal failure in rats. Nephron 44, 230–4 (1986). 3785486 10.1159/000183992

[b21] Peterson J. W., Boldogh I., Popov V. L., Saini S. S. & Chopra A. K. Anti-inflammatory and antisecretory potential of histidine in Salmonella-challenged mouse small intestine. Lab. Invest. 78, 523–34 (1998). 9605177

[b22] Watanabe M. *et al.* Consequences of low plasma histidine in chronic kidney disease patients: associations with inflammation, oxidative stress, and mortality. Am. J. Clin. Nutr. 87, 1860–6 (2008). 18541578 10.1093/ajcn/87.6.1860

[b23] Stockenhuber F., Aunder-Plassmann G. & Baleke P. Increased plasma histamine levels in chronic renal failure. N. Engl. J. Med. 317, 386 (1987). 3600734 10.1056/NEJM198708063170614

[b24] Gill D. S. *et al.* Plasma histamine in patients with chronic renal failure and nephrotic syndrome. J. Clin. Pathol. 44, 243–5 (1991). 2013627 10.1136/jcp.44.3.243 PMC496948

[b25] Choi J. Y. *et al.* Dialysis modality-dependent changes in serum metabolites: accumulation of inosine and hypoxanthine in patients on haemodialysis. Nephrol. Dial. Transplant 26, 1304–1313 (2011). 20844182 10.1093/ndt/gfq554

[b26] Posada-Ayala M. *et al.* Identification of a urine metabolomic signature in patients with advanced-stage chronic kidney disease. Kidney Int. 85, 103–11 (2014). 24048377 10.1038/ki.2013.328

[b27] Lameire N., Vanholder R. & De Smet R. Uremic toxins and peritoneal dialysis. Kidney Int. 59, S292–297 (2001). 10.1046/j.1523-1755.2001.59780292.x 11169029

[b28] Wong J. *et al.* Expansion of urease- and uricase-containing, indole- and p-cresol-forming and contraction of short-chain fatty acid-producing intestinal microbiota in ESRD. Am. J. Nephrol. 39, 230–7 (2014). 24643131 10.1159/000360010 PMC4049264

[b29] Wikoff W. R. *et al.* Metabolomics analysis reveals large effects of gut microflora on mammalian blood metabolites. Proc. Natl. Acad. Sci. 106, 3698–703 (2009). 19234110 10.1073/pnas.0812874106 PMC2656143

[b30] La S., Yoo H. H. & Kim D. H. Liquid chromatography-mass spectrometric analysis of urinary metabolites and their pattern recognition for the prediction of drug-induced hepatotoxicity. Chem. Res. Toxicol. 18, 1887–96 (2005). 16359179 10.1021/tx050187d

[b31] Vaziri N. D. *et al.* Chronic kidney disease alters intestinal microbial flora. Kidney Int 83, 308–15 (2013). 22992469 10.1038/ki.2012.345

[b32] Pawlak K., Kowalewska A., Mysliwiec M. & Pawlak D. Kynurenine and its metabolites–kynurenic acid and anthranilic acid are associated with soluble endothelial adhesion molecules and oxidative status in patients with chronic kidney disease. Am. J. Med. Sci. 338, 293–300 (2009). 19745702 10.1097/MAJ.0b013e3181aa30e6

[b33] Zhao J. Plasma kynurenic acid/tryptophan ratio: a sensitive and reliable biomarker for the assessment of renal function. Ren. Fail 35, 648–53 (2013). 23650931 10.3109/0886022X.2013.790301

[b34] Mutsaers H. A. *et al.* Uremic toxins inhibit transport by breast cancer resistance protein and multidrug resistance protein 4 at clinically relevant concentrations. PLoS One 6, e18438 (2011). 21483698 10.1371/journal.pone.0018438 PMC3070735

[b35] Byrd D. J. *et al.* Indolic tryptophan metabolism in uraemia. Proc. Eur. Dial. Transplant Assoc. 12, 347–54 (1976). 935125

[b36] Garibotto G. *et al.* Causes of hyperhomocysteinemia in patients with chronic kidney diseases. Semin. Nephrol. 26, 3–7 (2006). 16412817 10.1016/j.semnephrol.2005.06.002

[b37] McGregor D. O. *et al.* Dimethylglycine accumulates in uremia and predicts elevated plasma homocysteine concentrations. Kidney Int. 59, 2267–72 (2001). 11380830 10.1046/j.1523-1755.2001.00743.x

[b38] Skiba W. E., Taylor M. P., Wells M. S., Mangum J. H. & Awad W. M. Jr. Human hepatic methionine biosynthesis. Purification and characterization of betaine:homocysteine S-methyltransferase. J. Biol. Chem. 257, 14944–8 (1982). 7174675

[b39] Wyngaarden J. B. & Dunn J. T. 8-Hydroxyadenine as the intermediate in the oxidation of adenine to 2, 8-dihydroxyadenine by xanthine oxidase. Arch. Biochem. Biophys. 70, 150–6 (1957). 13445250 10.1016/0003-9861(57)90088-7

[b40] de Vries A. & Sperling O. Implications of disorders of purine metabolism for the kidney and the urinary tract. Ciba. Found. Symp. 48, 179–206 (1977). 24529 10.1002/9780470720301.ch12

[b41] Ayvazian J. H. & Skupp S. The Study of Purine Utilization and Excretion in a Xanthinuric Man. J. Clin. Invest. 44, 1248–60 (1965). 14328401 10.1172/JCI105231 PMC292599

[b42] Yamamoto T., Moriwaki Y. & Takahashi S. Effect of ethanol on metabolism of purine bases (hypoxanthine, xanthine, and uric acid). Clin. Chim. Acta 356, 35–57 (2005). 15936302 10.1016/j.cccn.2005.01.024

[b43] Shahbazian H., Zand Moghadam A., Ehsanpour A. & Khazaali M. Changes in plasma concentrations of hypoxanthine and uric acid before and after hemodialysis. Iran. J. Kidney Dis. 3, 151–5 (2009). 19617664

[b44] Spencer H. W., Yarger W. E. & Robinson R. R. Alterations of renal function during dietary-induced hyperuricemia in the rat. Kidney Int. 9, 489–500 (1976). 940282 10.1038/ki.1976.63

[b45] Jalal D. I., Chonchol M., Chen W. & Targher G. Uric acid as a target of therapy in CKD. Am. J. Kidney Dis. 61, 134–46 (2013). 23058478 10.1053/j.ajkd.2012.07.021 PMC3525781

[b46] Sanchez-Lozada L. G. *et al.* Effect of febuxostat on the progression of renal disease in 5/6 nephrectomy rats with and without hyperuricemia. Nephron. Physiol. 108, 69–78 (2008). 10.1159/000127837 18434753

[b47] Kosugi T. *et al.* Effect of lowering uric acid on renal disease in the type 2 diabetic db/db mice. Am. J. Physiol. Renal Physiol. 297, 481–8 (2009). 10.1152/ajprenal.00092.2009 PMC2724243 19458127

[b48] Suliman M. E., Barany P., Filho J. C., Lindholm B. & Bergstrom J. Accumulation of taurine in patients with renal failure. Nephrol. Dial. Transplant 17, 528–9 (2002). 11865115 10.1093/ndt/17.3.528

[b49] Bergstrom J., Alvestrand A., Furst P. & Lindholm B. Sulphur amino acids in plasma and muscle in patients with chronic renal failure: evidence for taurine depletion. J. Intern. Med. 226, 189–94 (1989). 2794849 10.1111/j.1365-2796.1989.tb01378.x

[b50] Fontana M., Pecci L., Dupre S. & Cavallini D. Antioxidant properties of sulfinates: protective effect of hypotaurine on peroxynitrite-dependent damage. Neurochem. Res. 29, 111–6 (2004). 14992269 10.1023/b:nere.0000010439.99991.cf

[b51] Hayashi K. *et al.* Use of serum and urine metabolome analysis for the detection of metabolic changes in patients with stage 1-2 chronic kidney disease. Nephro. Urol. Mon. 3, 164–171 (2011).

[b52] Bain M. A., Faull R., Fornasini G., Milne R. W. & Evans A. M. Accumulation of trimethylamine and trimethylamine-N-oxide in end-stage renal disease patients undergoing haemodialysis. Nephrol. Dial. Transplant 21, 1300–4 (2006). 16401621 10.1093/ndt/gfk056

[b53] Hauet T. *et al.* Influence of retrieval conditions on renal medulla injury: evaluation by proton NMR spectroscopy in an isolated perfused pig kidney model. J. Surg. Res. 93, 1–8 (2000). 10945936 10.1006/jsre.2000.5885

[b54] Hauet T., Gibelin H., Godart C., Eugene M. & Carretier M. Kidney retrieval conditions influence damage to renal medulla: evaluation by proton nuclear magnetic resonance (NMR) pectroscopy. Clin. Chem. Lab. Med. 38, 1085–92 (2000). 11156333 10.1515/CCLM.2000.161

[b55] Wang Z. *et al.* Gut flora metabolism of phosphatidylcholine promotes cardiovascular disease. Nature 472, 57–63 (2011). 21475195 10.1038/nature09922 PMC3086762

[b56] Zhao Y. Y. *et al.* A pharmaco-metabonomic study on chronic kidney disease and therapeutic effect of ergone by UPLC-QTOF/HDMS. Plos One 9, e115467 (2014). 25535749 10.1371/journal.pone.0115467 PMC4275224

[b57] Zhao Y. Y. *et al.* Intrarenal metabolomic investigation of chronic kidney disease and its TGF-beta1 mechanism in induced-adenine rats using UPLC Q-TOF/HSMS/MS^E^. J. Proteome. Res. 12, 692–703 (2013). 23227912 10.1021/pr3007792

[b58] Zhao Y. Y. *et al.* Ultra performance liquid chromatography-based metabonomic study of therapeutic effect of the surface layer of Poria cocos on adenine-induced chronic kidney disease provides new insight into anti-fibrosis mechanism. PLoS One 8, e59617 (2013). 23555727 10.1371/journal.pone.0059617 PMC3608665

[b59] Vaziri N. D. *et al.* High amylose resistant starch diet ameliorates oxidative stress, inflammation, and progression of chronic kidney disease. PLoS One 9, e114881 (2014). 25490712 10.1371/journal.pone.0114881 PMC4260945

[b60] Junqueira L. C., Bignolas G. & Brentani R. R. Picrosirius staining plus polarization microscopy, a specific method for collagen detection in tissue sections. Histochem. J. 11, 447–55 (1979). 91593 10.1007/BF01002772

[b61] Li L. *et al.* Total extract of Yupingfeng attenuates bleomycin-induced pulmonary fibrosis in rats. Phytomedicine 22, 111–9 (2015). 25636879 10.1016/j.phymed.2014.10.011

[b62] Jansson J. *et al.* Metabolomics reveals metabolic biomarkers of Crohn’s disease. PLoS One 4, e6386 (2009). 19636438 10.1371/journal.pone.0006386 PMC2713417

[b63] Benjamini Y. & Hochberg Y. controlling the false discovery rate-a practical and powerful approach to multiple testing. J. R. Stat. Soc. B 57, 289–300 (1995).

[b64] Storey J. D. A direct approach to false discovery rates. J. R. Stat. Soc. B 2002, 479–498 (2002).

