# Supplementary material for: Metabolomics insights into chronic kidney disease and modulatory effect of rhubarb against tubulointerstitial fibrosis
Source: Sci Rep. 2015 Sep 28;5:14472. doi: 10.1038/srep14472 (PMC4585987; doi:10.1038/srep14472)
Supplement: Supplementary Information [file srep14472-s1.doc]

*Supporting Information*

[**Metabolomics insights into**](http://apps.webofknowledge.com/full_record.do?product=UA&search_mode=GeneralSearch&qid=29&SID=3FOryKcyoWerqL9ek29&page=1&doc=13&cacheurlFromRightClick=no) **chronic kidney disease and modulatory effect of rhubarb against tubulointerstitial fibrosis**

Zhi-Hao Zhang^3^, Feng Wei^4^, Nosratola D. Vaziri^2^, Xian-Long Cheng^4^, Xu Bai^6^, Rui-Chao Lin^5^, Ying-Yong Zhao^1,2,*^

^1^ Key Laboratory of Resource Biology and Biotechnology in Western China, Ministry of Education, the College of Life Sciences, Northwest University, No. 229 Taibai North Road, Xi’an, Shaanxi 710069, China

^2^ Division of Nephrology and Hypertension, School of Medicine, University of California, Irvine, MedSci 1, C352, UCI Campus, Irvine, California, 92697, USA

^3^National Center for Natural Products Research, Department of BioMolecular Sciences, School of Pharmacy, University of Mississippi, Oxford, Mississippi, 38677, USA

^4^ National Institutes for Food and Drug Control, State Food and Drug Administration, No. 2 Tiantan Xili, Beijing, 100050, China

^5^School of Chinese Materia Medica, Beijing University of Chinese Medicine, No. 11 North Third Ring Road, Beijing 100029, China

^6^Solution Centre, Waters Technologies (Shanghai) Ltd., No. 1000 Jinhai Road, Shanghai 201203, China

Corresponding author:

Professor Ying-Yong Zhao, PhD, MD, Tel: +86 29 88304569; Fax: +86 29 88304368; E-mail: zyy@nwu.edu.cn; zhaoyybr@163.com (Y.Y. Zhao)

UPLC conditions

Metabolite separation was performed using a Waters Acquity™ Ultra Performance LC system (Waters, USA) equipped with a Waters Xevo™ G2 QTof MS (Waters MS Technologies, Manchester, UK). Chromatographic separation was carried out at 45℃ on an ACQUITY UPLC HSS T3 column (2.1 mm, 100 mm, 1.8 mm, UK). The mobile phase consisted of water (A) and acetonitrile (B), each containing 0.1% formic acid. The optimized UPLC elution conditions were: 0–1.0 min, 1% B; 1.0–8.0 min, 1–40% B; 8.0–9.0 min, 40–99% B; 9.0–12.0 min, 99% B and 12.0–15.0 min, 99.0–1.0% B. The ﬂow rate was 0.40 mL/min. The autosampler was maintained at 4℃. Every 2 mL sample solution was injected for each run.

Mass spectrometry

Mass spectrometry was performed using a Xevo™ G2 QT of (Waters MS Technologies, Manchester, UK), a quadrupole and orthogonal acceleration time-of-ﬂight tandem mass spectrometer. The scan range was from 50 to 1000 m/z. For positive electrospray modes, the capillary and cone voltage were set at 3.0 kV and 40 V, respectively. The desolvation gas was set to 800 L/h at a temperature of 450 1C, the cone gas was set to 50 L/h and the source temperature was set to 120 1C. The mass spectrometer was operated in the W optics mode with 12,000 resolution using dynamic range extension. The data acquisition rate was set to 0.1 s, with a 0.1 s interscan delay. All analyses were acquired using the lockspray to ensure accuracy and reproducibility. All of the data acquisition and analyses of data were controlled by Waters MassLynx v4.1 software.

Table S1. Differentially expressed metabolites between the Control and CKD model groups in week 3.

|  | CKD VS Control | | | | |  | EA VS Control | | |  | EA VS CKD | | |  | BU VS Control | | |  | BU VS CKD | | |  | PE VS Control | | |  | PE VS CKD | | |
| --- | --- | --- | --- | --- | --- | --- | --- | --- | --- | --- | --- | --- | --- | --- | --- | --- | --- | --- | --- | --- | --- | --- | --- | --- | --- | --- | --- | --- | --- |
| Metabolites | VIP | p^a^ | p^b^ | FDR^c^ | FC^d^ |  | p^a^ | p^b^ | FC^d^ |  | p^a^ | p^b^ | FC^d^ |  | p^a^ | p^b^ | FC^d^ |  | p^a^ | p^b^ | FC^d^ |  | p^a^ | p^b^ | FC^d^ |  | p^a^ | p^b^ | FC^d^ |
| 1-Phenylethylamine | 3.65 | 2.76E-11 | 9.63E-07 | 6.50E-11 | -3.18 |  | 1.27E-11 | 5.16E-07 | -6.65 |  | 2.53E-02 | 1.32E-02 | -2.94 |  | 3.69E-12 | 1.71E-09 | -7.69 |  | 3.71E-11 | 9.12E-07 | -3.45 |  | 7.53E-11 | 9.63E-07 | -2.74 |  | 1.07E-02 | 5.62E-02 | 0.47 |
| 2,8-Dihydroxyadenine | 8.79 | 1.60E-08 | 1.87E-06 | 2.43E-08 | 6.04 |  | 8.39E-14 | 3.46E-07 | 6.84 |  | 5.54E-02 | 1.95E-02 | 0.78 |  | 2.63E-16 | 1.71E-09 | 7.19 |  | 1.35E-01 | 7.76E-02 | 0.67 |  | 9.13E-16 | 3.46E-07 | 6.85 |  | 4.01E-02 | 3.36E-02 | 0.80 |
| 2-Keto-glutaramic acid | 3.69 | 2.12E-03 | 1.77E-04 | 2.18E-03 | 3.52 |  | 1.46E-01 | 5.70E-04 | 2.79 |  | 1.37E-02 | 1.08E-02 | -0.77 |  | 2.49E-03 | 6.79E-05 | 3.63 |  | 1.54E-01 | 7.73E-01 | 0.03 |  | 4.46E-02 | 1.77E-04 | 3.07 |  | 2.08E-01 | 1.17E-02 | -0.48 |
| 2-Methylpropanal | 3.19 | 1.27E-11 | 9.63E-07 | 3.32E-11 | 1.56 |  | 1.11E-07 | 2.73E-06 | 1.03 |  | 2.07E-04 | 1.57E-04 | -0.56 |  | 1.09E-10 | 1.71E-09 | 1.60 |  | 9.69E-01 | 8.01E-01 | 0.00 |  | 4.26E-11 | 9.63E-07 | 1.29 |  | 1.28E-02 | 1.05E-02 | -0.30 |
| 2-Pentenal | 2.97 | 3.91E-09 | 9.63E-07 | 6.49E-09 | 2.97 |  | 2.00E-06 | 7.61E-06 | 1.49 |  | 1.12E-04 | 6.90E-06 | -1.51 |  | 8.41E-03 | 3.35E-01 | -0.14 |  | 3.27E-04 | 8.39E-05 | -2.92 |  | 5.72E-03 | 6.57E-02 | -0.18 |  | 1.15E-05 | 7.67E-06 | -3.04 |
| 3-Dehydrocarnitine | 4.09 | 1.94E-16 | 9.63E-07 | 1.77E-15 | 2.60 |  | 1.77E-17 | 1.71E-09 | 2.21 |  | 2.51E-04 | 6.53E-04 | -0.41 |  | 6.64E-10 | 1.71E-09 | 2.10 |  | 1.82E-04 | 4.76E-04 | -0.66 |  | 3.27E-15 | 9.63E-07 | 2.16 |  | 1.65E-04 | 5.44E-04 | -0.47 |
| 3-Methyldioxyindole | 6.11 | 3.52E-05 | 1.16E-04 | 4.22E-05 | 1.04 |  | 6.20E-01 | 1.60E-01 | 0.13 |  | 3.95E-05 | 1.41E-04 | -0.97 |  | 7.92E-01 | 8.95E-01 | -0.23 |  | 8.88E-05 | 5.82E-03 | -1.42 |  | 1.68E-02 | 4.07E-02 | 0.49 |  | 1.62E-03 | 5.82E-03 | -0.58 |
| 3-Methylene-indolenine | 8.11 | 3.81E-09 | 2.83E-06 | 6.47E-09 | -1.39 |  | 2.05E-02 | 3.36E-02 | -0.34 |  | 2.66E-06 | 3.99E-05 | 1.11 |  | 2.61E-08 | 1.71E-09 | -0.95 |  | 3.41E-02 | 3.82E-02 | 0.49 |  | 2.03E-09 | 9.63E-07 | -1.03 |  | 1.29E-01 | 3.82E-02 | 0.38 |
| 3-Methylhistidine | 3.73 | 3.84E-16 | 9.63E-07 | 3.11E-15 | -1.99 |  | 8.65E-18 | 9.63E-07 | -1.43 |  | 2.97E-01 | 9.73E-02 | 0.54 |  | 1.03E-17 | 1.71E-09 | -2.67 |  | 4.07E-01 | 3.68E-01 | -0.46 |  | 1.82E-17 | 9.63E-07 | -1.17 |  | 1.19E-02 | 2.11E-02 | 0.81 |
| 3-Methyluridine | 2.00 | 7.25E-15 | 9.63E-07 | 4.41E-14 | 1.34 |  | 8.02E-03 | 1.15E-02 | -1.96 |  | 1.39E-13 | 1.34E-06 | -3.20 |  | 2.74E-02 | 3.29E-02 | -0.95 |  | 3.56E-14 | 9.61E-07 | -2.18 |  | 1.91E-08 | 2.83E-06 | 1.17 |  | 1.43E-01 | 1.43E-02 | -0.18 |
| 3-O-Methyldopa | 2.33 | 1.54E-02 | 5.18E-02 | 1.54E-02 | -0.32 |  | 1.49E-02 | 3.36E-02 | -0.26 |  | 8.43E-01 | 5.22E-01 | 0.06 |  | 1.31E-05 | 1.53E-05 | -1.46 |  | 6.83E-04 | 1.79E-04 | -0.96 |  | 1.70E-02 | 5.62E-02 | -0.24 |  | 7.05E-01 | 8.57E-01 | 0.09 |
| 4-Aminohippuric acid | 3.38 | 1.69E-10 | 1.11E-05 | 3.62E-10 | -3.65 |  | 1.66E-07 | 1.54E-05 | -1.69 |  | 8.00E-06 | 8.87E-05 | 2.02 |  | 6.74E-09 | 4.66E-07 | -2.45 |  | 7.13E-02 | 7.28E-03 | 0.90 |  | 1.51E-08 | 5.62E-02 | -3.27 |  | 3.98E-02 | 7.32E-02 | 0.52 |
| 4-Hydroxyproline | 2.00 | 2.38E-13 | 4.30E-07 | 8.69E-13 | -5.64 |  | 1.54E-15 | 9.63E-07 | 1.41 |  | 5.91E-09 | 1.68E-06 | 1.92 |  | 1.09E-08 | 1.93E-05 | 1.24 |  | 5.12E-06 | 1.43E-04 | 1.61 |  | 6.89E-08 | 7.42E-05 | 1.26 |  | 1.98E-05 | 9.16E-04 | 1.77 |
| 5-Aminoimidazole | 3.64 | 3.48E-12 | 7.47E-07 | 9.41E-12 | -5.50 |  | 2.14E-10 | 2.06E-08 | -3.95 |  | 1.16E-02 | 2.60E-02 | 1.46 |  | 3.70E-11 | 5.14E-08 | -5.67 |  | 2.78E-01 | 4.67E-01 | 0.23 |  | 2.60E-10 | 2.82E-06 | -2.65 |  | 1.47E-04 | 3.22E-04 | 2.59 |
| 8-Hydroxyguanine | 8.79 | 1.56E-11 | 7.47E-07 | 3.92E-11 | 8.76 |  | 2.99E-18 | 7.47E-07 | 9.28 |  | 4.43E-03 | 6.66E-03 | 0.56 |  | 1.67E-17 | 1.71E-09 | 9.47 |  | 3.46E-02 | 9.11E-04 | 0.43 |  | 1.53E-16 | 7.47E-07 | 9.43 |  | 3.54E-04 | 9.11E-04 | 0.71 |
| Acetylcysteine | 2.82 | 6.36E-06 | 9.02E-06 | 8.29E-06 | -4.54 |  | 5.08E-01 | 8.73E-01 | 0.35 |  | 8.01E-04 | 3.33E-09 | 4.63 |  | 7.13E-04 | 2.68E-03 | 0.94 |  | 1.53E-15 | 7.47E-07 | 5.37 |  | 8.67E-01 | 2.80E-01 | -0.68 |  | 1.40E-03 | 1.00E-04 | 3.66 |
| Adenine | 2.30 | 3.15E-05 | 2.07E-04 | 3.83E-05 | 1.71 |  | 8.92E-02 | 2.53E-02 | 0.84 |  | 3.83E-04 | 6.53E-04 | -0.93 |  | 1.07E-02 | 8.68E-03 | 0.82 |  | 3.97E-03 | 4.96E-03 | -0.94 |  | 4.45E-02 | 9.50E-03 | 0.95 |  | 1.90E-02 | 1.17E-02 | -0.81 |
| Asparagine | 6.69 | 8.54E-19 | 9.63E-07 | 2.08E-17 | 4.28 |  | 9.77E-09 | 9.63E-07 | 2.92 |  | 5.22E-11 | 1.41E-06 | -1.44 |  | 1.51E-11 | 1.71E-09 | 3.05 |  | 1.19E-12 | 2.96E-06 | -1.45 |  | 2.94E-13 | 9.63E-07 | 3.35 |  | 2.51E-09 | 2.96E-06 | -0.99 |
| Citrulline | 3.37 | 1.19E-14 | 9.61E-07 | 6.22E-14 | 2.95 |  | 5.23E-03 | 2.07E-02 | 0.79 |  | 4.18E-09 | 3.33E-09 | -2.10 |  | 5.76E-07 | 2.48E-04 | 2.41 |  | 1.38E-12 | 9.63E-07 | -0.79 |  | 3.32E-05 | 4.75E-04 | 1.98 |  | 6.39E-15 | 9.63E-07 | -1.02 |
| Creatine | 4.39 | 1.88E-03 | 2.37E-06 | 1.96E-03 | 1.90 |  | 8.27E-04 | 7.71E-08 | 2.13 |  | 5.73E-01 | 5.90E-01 | 0.24 |  | 7.88E-04 | 3.43E-09 | 1.65 |  | 1.77E-01 | 7.73E-01 | -0.44 |  | 1.82E-05 | 1.16E-06 | 2.28 |  | 7.21E-01 | 5.64E-01 | 0.40 |
| Creatinine | 2.87 | 3.59E-05 | 2.93E-05 | 4.23E-05 | 0.43 |  | 7.70E-05 | 5.44E-04 | 0.44 |  | 6.10E-09 | 2.96E-06 | 0.92 |  | 5.14E-05 | 1.00E-04 | 0.42 |  | 3.23E-07 | 1.09E-05 | 0.87 |  | 1.31E-01 | 4.49E-01 | 0.15 |  | 6.13E-04 | 6.93E-03 | 0.60 |
| Cytosine | 3.44 | 2.05E-06 | 1.66E-06 | 2.72E-06 | -2.33 |  | 1.10E-05 | 4.66E-07 | -1.73 |  | 1.65E-01 | 1.96E-01 | 0.58 |  | 2.15E-05 | 7.71E-08 | -1.38 |  | 1.21E-02 | 4.37E-02 | 1.09 |  | 3.15E-06 | 9.63E-07 | -1.94 |  | 6.62E-01 | 8.57E-01 | 0.36 |
| Deoxyadenosine | 2.96 | 1.61E-12 | 3.46E-07 | 4.71E-12 | -7.18 |  | 6.14E-03 | 2.29E-02 | -1.12 |  | 4.79E-10 | 5.29E-06 | 5.66 |  | 6.49E-09 | 1.71E-09 | -2.67 |  | 9.32E-07 | 6.34E-06 | 4.25 |  | 1.87E-11 | 5.16E-07 | -6.12 |  | 9.03E-02 | 1.58E-01 | 0.98 |
| Dihydrouracil | 2.95 | 5.85E-17 | 8.95E-07 | 6.10E-16 | 5.09 |  | 8.53E-09 | 2.99E-05 | 3.62 |  | 1.30E-05 | 1.87E-05 | -1.44 |  | 5.74E-05 | 1.71E-09 | 2.18 |  | 2.85E-06 | 1.27E-05 | -2.63 |  | 4.00E-13 | 8.95E-07 | 4.79 |  | 3.42E-02 | 1.43E-02 | -0.33 |
| Dimethylglycine | 3.62 | 1.14E-09 | 6.71E-06 | 2.13E-09 | 1.03 |  | 5.20E-03 | 1.92E-02 | -0.44 |  | 1.14E-12 | 1.41E-06 | -1.56 |  | 3.81E-01 | 5.57E-01 | -0.14 |  | 5.43E-04 | 2.79E-03 | -1.32 |  | 9.20E-01 | 5.40E-01 | -0.21 |  | 1.45E-06 | 2.50E-05 | -1.31 |
| Dopamine | 4.05 | 7.66E-08 | 6.71E-06 | 1.08E-07 | -1.15 |  | 8.56E-04 | 1.52E-03 | -0.44 |  | 2.31E-03 | 5.94E-03 | 0.75 |  | 1.69E-05 | 6.39E-07 | -0.53 |  | 2.79E-03 | 2.32E-02 | 0.71 |  | 9.33E-09 | 9.39E-06 | -1.02 |  | 8.06E-01 | 8.85E-01 | 0.13 |
| D-Xylulose | 2.58 | 8.57E-06 | 8.56E-07 | 1.10E-05 | 5.24 |  | 3.00E-05 | 2.48E-03 | 2.75 |  | 2.47E-03 | 3.92E-04 | -2.40 |  | 1.87E-03 | 6.39E-07 | 1.75 |  | 2.19E-03 | 5.83E-03 | -3.55 |  | 1.02E-05 | 8.83E-06 | 4.28 |  | 1.09E-01 | 4.76E-02 | -0.95 |
| Ethyl-N2-acetyl-L-argininate | 3.59 | 2.36E-08 | 1.31E-05 | 3.51E-08 | -1.19 |  | 4.09E-10 | 9.63E-07 | -1.03 |  | 6.81E-01 | 5.00E-02 | 0.17 |  | 4.99E-10 | 1.71E-09 | -1.20 |  | 9.18E-01 | 5.17E-01 | 0.05 |  | 3.94E-09 | 9.63E-07 | -0.88 |  | 1.63E-01 | 2.37E-02 | 0.33 |
| Glyceric acid | 3.85 | 7.89E-14 | 9.63E-07 | 3.39E-13 | 2.55 |  | 1.20E-14 | 9.63E-07 | 2.31 |  | 5.29E-02 | 7.04E-02 | -0.26 |  | 8.35E-11 | 1.71E-09 | 2.81 |  | 5.51E-01 | 7.73E-01 | 0.04 |  | 8.05E-12 | 9.63E-07 | 2.54 |  | 8.66E-01 | 9.40E-01 | -0.01 |
| Glycolic acid | 2.98 | 1.41E-03 | 8.50E-05 | 1.51E-03 | -4.03 |  | 8.20E-01 | 4.44E-01 | 1.05 |  | 2.00E-06 | 9.98E-08 | 4.89 |  | 8.08E-01 | 4.61E-01 | 0.27 |  | 5.22E-07 | 4.29E-05 | 4.53 |  | 3.16E-02 | 2.82E-02 | -2.72 |  | 3.63E-02 | 2.17E-01 | 1.32 |
| Glycyl-Arginine | 2.32 | 2.55E-14 | 1.96E-07 | 1.24E-13 | 6.57 |  | 3.00E-06 | 1.45E-04 | 4.40 |  | 6.70E-05 | 5.73E-05 | -2.06 |  | 9.39E-16 | 1.71E-09 | 6.77 |  | 1.01E-01 | 8.38E-02 | -0.24 |  | 1.63E-13 | 1.96E-07 | 5.51 |  | 8.11E-07 | 1.04E-05 | -1.13 |
| Glycyl-Lysine | 4.34 | 3.96E-09 | 5.66E-06 | 6.43E-09 | 2.16 |  | 1.00E-05 | 1.74E-04 | 1.71 |  | 4.00E-06 | 1.47E-05 | -0.48 |  | 3.31E-07 | 2.94E-04 | 2.47 |  | 4.07E-01 | 1.13E-01 | -0.13 |  | 1.16E-05 | 5.44E-04 | 1.73 |  | 2.65E-05 | 1.21E-04 | -0.46 |
| Glycylproline | 3.60 | 3.41E-10 | 2.67E-07 | 6.72E-10 | 7.94 |  | 2.19E-01 | 8.49E-01 | 0.23 |  | 9.45E-10 | 4.68E-07 | -7.31 |  | 7.44E-02 | 4.20E-01 | 0.63 |  | 1.05E-09 | 5.16E-07 | -7.05 |  | 9.27E-05 | 9.59E-04 | 1.83 |  | 1.31E-09 | 1.30E-06 | -6.10 |
| Glyoxylic acid | 3.08 | 2.29E-11 | 9.63E-07 | 5.57E-11 | -2.01 |  | 4.78E-10 | 1.20E-08 | -1.36 |  | 2.61E-02 | 4.26E-02 | 0.68 |  | 1.48E-10 | 1.71E-09 | -2.46 |  | 4.06E-01 | 4.07E-01 | -0.12 |  | 5.51E-12 | 9.55E-07 | -3.13 |  | 2.95E-01 | 4.29E-01 | -1.01 |
| Histamine | 2.10 | 4.40E-13 | 9.24E-07 | 1.40E-12 | -3.29 |  | 2.36E-18 | 3.46E-07 | -6.10 |  | 2.20E-02 | 5.88E-03 | -2.43 |  | 1.04E-24 | 1.71E-09 | -6.98 |  | 1.13E-03 | 4.79E-04 | -2.83 |  | 5.45E-18 | 9.43E-07 | -3.08 |  | 6.47E-01 | 8.79E-01 | 0.17 |
| Homocitrulline | 4.60 | 1.20E-17 | 8.95E-07 | 1.75E-16 | 6.77 |  | 8.28E-08 | 1.71E-09 | 5.12 |  | 2.83E-10 | 9.98E-08 | -1.75 |  | 1.27E-07 | 1.71E-09 | 4.05 |  | 9.75E-13 | 9.61E-07 | -2.79 |  | 7.76E-07 | 8.95E-07 | 5.80 |  | 7.98E-04 | 1.19E-03 | -1.03 |
| Homocysteine | 10.26 | 4.12E-13 | 9.63E-07 | 1.37E-12 | -1.74 |  | 2.44E-11 | 1.99E-06 | -1.24 |  | 1.41E-02 | 1.95E-02 | 0.53 |  | 3.57E-10 | 3.43E-09 | -1.21 |  | 2.53E-03 | 1.34E-02 | 0.68 |  | 9.17E-13 | 1.16E-06 | -1.57 |  | 4.46E-01 | 5.98E-01 | 0.18 |
| Homo-L-arginine | 2.83 | 4.88E-19 | 5.16E-07 | 1.78E-17 | 6.63 |  | 5.75E-17 | 1.71E-09 | 7.01 |  | 1.97E-03 | 1.36E-02 | 0.40 |  | 1.14E-17 | 1.71E-09 | 7.44 |  | 9.54E-03 | 4.00E-02 | 0.32 |  | 2.75E-18 | 5.16E-07 | 7.11 |  | 1.21E-04 | 5.51E-05 | 0.51 |
| Hydroxypyruvic acid | 4.27 | 2.24E-03 | 8.61E-05 | 2.27E-03 | -3.09 |  | 5.79E-01 | 5.17E-01 | 0.76 |  | 5.44E-07 | 2.46E-06 | 3.95 |  | 9.39E-01 | 5.09E-01 | 0.41 |  | 4.19E-07 | 2.13E-05 | 3.92 |  | 2.96E-02 | 1.40E-02 | -3.21 |  | 4.81E-02 | 6.76E-01 | 0.12 |
| Hypoxanthine | 2.96 | 6.20E-09 | 3.37E-06 | 9.63E-09 | -1.92 |  | 4.75E-08 | 1.66E-07 | -1.51 |  | 1.54E-01 | 1.10E-01 | 0.44 |  | 5.35E-09 | 1.71E-09 | -1.30 |  | 1.38E-01 | 1.40E-01 | 0.72 |  | 1.02E-09 | 9.55E-07 | -2.60 |  | 5.71E-01 | 8.06E-01 | -0.60 |
| Indole-3-carboxylic acid | 4.08 | 5.99E-09 | 9.63E-07 | 9.50E-09 | -1.16 |  | 5.99E-09 | 1.71E-09 | -1.20 |  | 5.89E-01 | 4.91E-01 | -0.05 |  | 1.41E-11 | 1.71E-09 | -1.80 |  | 2.83E-03 | 3.14E-03 | -0.50 |  | 9.22E-11 | 9.63E-07 | -1.53 |  | 3.22E-02 | 5.00E-02 | -0.40 |
| Kynurenic acid | 4.38 | 3.71E-09 | 9.63E-07 | 6.45E-09 | -1.32 |  | 1.78E-09 | 1.71E-09 | -1.39 |  | 5.77E-01 | 5.90E-01 | -0.07 |  | 7.21E-11 | 1.71E-09 | -2.00 |  | 8.38E-04 | 1.19E-03 | -0.51 |  | 2.21E-10 | 9.63E-07 | -1.70 |  | 1.59E-02 | 1.95E-02 | -0.40 |
| Kynurenine | 3.94 | 9.78E-15 | 5.16E-07 | 5.49E-14 | -7.71 |  | 1.48E-14 | 8.07E-07 | -6.51 |  | 1.38E-02 | 6.58E-03 | 1.01 |  | 1.24E-14 | 1.71E-09 | -7.42 |  | 9.04E-02 | 1.44E-01 | 0.55 |  | 1.17E-14 | 5.99E-07 | -7.27 |  | 1.71E-01 | 4.19E-01 | 0.39 |
| L-Acetylcarnitine | 5.12 | 3.19E-09 | 9.63E-07 | 5.69E-09 | 3.60 |  | 1.12E-06 | 1.79E-04 | 1.90 |  | 1.91E-04 | 6.47E-05 | -1.75 |  | 1.68E-03 | 2.16E-03 | 0.18 |  | 6.73E-04 | 2.23E-07 | -3.32 |  | 5.58E-05 | 2.16E-03 | 1.50 |  | 2.82E-05 | 2.23E-07 | -2.18 |
| L-Carnitine | 7.26 | 2.55E-13 | 9.63E-07 | 8.88E-13 | 3.88 |  | 9.99E-08 | 4.01E-06 | 2.89 |  | 5.46E-04 | 2.00E-03 | -1.05 |  | 4.85E-06 | 2.73E-06 | 2.52 |  | 5.24E-07 | 1.04E-04 | -1.59 |  | 1.31E-06 | 2.13E-05 | 2.55 |  | 4.12E-05 | 1.04E-04 | -1.42 |
| Leucyl-Proline | 3.10 | 2.39E-05 | 2.13E-05 | 2.96E-05 | 1.59 |  | 1.47E-08 | 6.39E-07 | 1.68 |  | 7.22E-01 | 1.02E-01 | 0.10 |  | 2.06E-10 | 4.62E-06 | 1.76 |  | 5.01E-01 | 4.76E-02 | 0.16 |  | 9.52E-07 | 2.93E-05 | 1.57 |  | 8.39E-01 | 4.07E-01 | -0.01 |
| L-Gulose | 3.37 | 7.65E-13 | 9.63E-07 | 2.33E-12 | -2.50 |  | 7.67E-01 | 8.01E-01 | -0.06 |  | 2.07E-09 | 1.41E-06 | 2.53 |  | 6.96E-04 | 2.99E-05 | -0.69 |  | 4.34E-05 | 1.55E-04 | 1.84 |  | 1.14E-05 | 1.00E-04 | -0.84 |  | 2.75E-05 | 5.51E-05 | 1.70 |
| L-Histidine | 4.52 | 1.23E-17 | 9.63E-07 | 1.49E-16 | -2.93 |  | 1.73E-16 | 1.71E-09 | -3.49 |  | 1.91E-01 | 6.15E-02 | -0.41 |  | 1.18E-17 | 1.71E-09 | -6.11 |  | 3.73E-02 | 3.54E-02 | -2.45 |  | 1.36E-16 | 9.63E-07 | -2.44 |  | 9.26E-03 | 2.62E-02 | 0.53 |
| L-Phenylalanine | 3.17 | 1.71E-18 | 9.63E-07 | 3.13E-17 | -1.64 |  | 5.24E-21 | 1.71E-09 | -1.37 |  | 1.06E-01 | 5.13E-02 | 0.29 |  | 2.27E-20 | 1.71E-09 | -2.11 |  | 5.26E-01 | 8.01E-01 | -0.32 |  | 1.21E-19 | 9.63E-07 | -1.39 |  | 1.41E-01 | 6.48E-02 | 0.27 |
| Melatonin | 3.22 | 9.24E-11 | 8.56E-07 | 2.04E-10 | -4.72 |  | 3.00E-06 | 4.66E-07 | -1.07 |  | 1.50E-05 | 2.36E-05 | 3.44 |  | 5.14E-10 | 1.71E-09 | -3.47 |  | 1.86E-01 | 2.12E-01 | 1.35 |  | 4.18E-11 | 8.07E-07 | -4.86 |  | 9.70E-01 | 8.74E-01 | -0.08 |
| Metenamine | 3.01 | 4.27E-05 | 5.47E-05 | 4.95E-05 | 1.58 |  | 1.20E-05 | 2.41E-05 | 1.22 |  | 5.57E-02 | 1.60E-01 | -0.39 |  | 1.33E-01 | 6.70E-02 | 0.55 |  | 1.92E-04 | 5.44E-04 | -1.20 |  | 3.97E-04 | 4.15E-04 | 1.29 |  | 4.35E-01 | 4.98E-01 | -0.31 |
| Methyladenine | 2.66 | 1.01E-04 | 7.00E-07 | 1.15E-04 | -6.03 |  | 1.53E-04 | 5.94E-06 | -4.89 |  | 4.26E-02 | 2.39E-01 | 0.96 |  | 1.20E-04 | 1.15E-07 | -6.00 |  | 8.54E-02 | 4.21E-02 | 0.54 |  | 1.08E-04 | 1.07E-06 | -5.79 |  | 3.25E-01 | 3.17E-01 | 0.19 |
| Methylcytosine | 2.92 | 1.49E-04 | 4.76E-04 | 1.68E-04 | -1.10 |  | 2.76E-01 | 9.01E-01 | 0.13 |  | 9.92E-04 | 1.90E-04 | 1.30 |  | 8.97E-01 | 9.85E-01 | -0.28 |  | 1.13E-02 | 5.62E-02 | 0.91 |  | 5.77E-02 | 1.74E-02 | -0.69 |  | 2.01E-01 | 2.43E-01 | 0.44 |
| N-Acetylleucine | 3.47 | 1.92E-05 | 2.83E-06 | 2.42E-05 | -1.66 |  | 2.00E-06 | 5.14E-08 | -3.30 |  | 4.48E-04 | 5.73E-05 | -1.55 |  | 3.12E-07 | 1.71E-09 | -6.39 |  | 5.06E-09 | 8.16E-07 | -4.17 |  | 1.17E-06 | 9.63E-07 | -2.93 |  | 6.70E-08 | 4.26E-06 | -1.28 |
| N-Acryloylglycine | 3.69 | 4.63E-11 | 1.96E-07 | 1.06E-10 | -7.65 |  | 1.74E-09 | 5.14E-08 | -3.37 |  | 2.00E-06 | 2.36E-05 | 3.73 |  | 6.26E-10 | 1.71E-09 | -5.32 |  | 1.23E-03 | 1.01E-02 | 2.38 |  | 1.33E-10 | 5.16E-07 | -6.39 |  | 4.41E-02 | 3.59E-02 | 1.09 |
| Nicotinic acid | 6.29 | 2.33E-19 | 9.63E-07 | 1.70E-17 | -1.65 |  | 1.45E-21 | 9.63E-07 | -1.44 |  | 2.10E-01 | 5.95E-02 | 0.22 |  | 7.18E-24 | 1.71E-09 | -1.83 |  | 3.70E-01 | 6.71E-02 | -0.06 |  | 2.43E-20 | 1.71E-09 | -1.41 |  | 1.34E-01 | 6.71E-02 | 0.25 |
| Phosphoric acid | 4.59 | 5.29E-14 | 9.63E-07 | 2.41E-13 | 1.41 |  | 2.43E-14 | 1.71E-09 | 1.45 |  | 5.29E-01 | 4.23E-01 | 0.05 |  | 1.42E-15 | 1.71E-09 | 1.81 |  | 8.90E-03 | 3.67E-02 | 0.21 |  | 5.65E-18 | 9.63E-07 | 1.60 |  | 3.70E-03 | 4.70E-03 | 0.20 |
| Pipecolic acid | 4.29 | 1.10E-13 | 9.63E-07 | 4.46E-13 | 0.92 |  | 2.37E-11 | 1.71E-09 | 0.68 |  | 9.56E-04 | 1.68E-03 | -0.26 |  | 9.88E-09 | 1.71E-09 | 0.91 |  | 6.34E-01 | 2.21E-01 | -0.07 |  | 4.16E-10 | 9.63E-07 | 0.92 |  | 8.86E-01 | 8.80E-01 | 0.00 |
| Prolinebetaine | 4.89 | 2.32E-13 | 9.63E-07 | 8.92E-13 | 1.09 |  | 1.71E-13 | 9.63E-07 | 0.91 |  | 5.23E-03 | 9.55E-03 | -0.19 |  | 3.18E-18 | 1.71E-09 | 1.18 |  | 5.89E-01 | 4.68E-01 | 0.04 |  | 2.76E-12 | 1.71E-09 | 1.03 |  | 4.53E-01 | 4.68E-01 | -0.06 |
| Purine | 4.22 | 4.46E-10 | 1.99E-06 | 8.57E-10 | 1.75 |  | 3.00E-05 | 1.45E-04 | 0.91 |  | 2.90E-05 | 2.36E-05 | -0.90 |  | 2.86E-06 | 2.99E-05 | 1.28 |  | 1.15E-04 | 8.62E-05 | -0.75 |  | 6.41E-07 | 1.00E-04 | 1.17 |  | 2.33E-03 | 2.91E-03 | -0.62 |
| Pyridoxine | 2.74 | 2.16E-09 | 9.63E-07 | 3.93E-09 | 1.30 |  | 4.93E-02 | 5.79E-02 | -0.97 |  | 3.23E-10 | 3.33E-09 | -2.28 |  | 5.36E-01 | 9.01E-01 | -1.77 |  | 1.50E-06 | 5.39E-05 | -2.88 |  | 9.37E-01 | 8.85E-01 | 0.04 |  | 2.73E-09 | 1.41E-06 | -1.34 |
| Pyrimidine | 3.24 | 2.74E-08 | 5.66E-06 | 4.01E-08 | 0.86 |  | 1.00E-06 | 1.17E-06 | 0.85 |  | 9.38E-01 | 1.00E+00 | -0.01 |  | 4.89E-12 | 2.16E-03 | 1.27 |  | 3.70E-05 | 1.19E-03 | 0.43 |  | 9.39E-04 | 2.79E-03 | 0.62 |  | 1.44E-01 | 3.09E-01 | -0.26 |
| Succinyladenosine | 3.21 | 1.99E-12 | 5.16E-07 | 5.58E-12 | -7.25 |  | 8.14E-02 | 1.46E-01 | -1.05 |  | 1.52E-09 | 8.94E-06 | 5.92 |  | 1.99E-08 | 1.71E-09 | -2.84 |  | 3.48E-06 | 1.94E-05 | 4.22 |  | 4.96E-11 | 5.16E-07 | -6.26 |  | 6.95E-02 | 6.92E-01 | 1.02 |
| Taurine | 3.55 | 1.25E-06 | 1.35E-05 | 1.68E-06 | -6.21 |  | 4.52E-07 | 2.34E-06 | -7.28 |  | 1.39E-01 | 7.37E-02 | -0.94 |  | 4.55E-07 | 1.57E-06 | -7.68 |  | 1.40E-01 | 6.56E-02 | -0.94 |  | 4.55E-07 | 2.43E-02 | -7.28 |  | 1.40E-01 | 7.37E-02 | -0.94 |
| Tryptophan | 4.25 | 8.91E-04 | 1.79E-04 | 9.71E-04 | 1.48 |  | 3.66E-01 | 2.53E-02 | 0.58 |  | 2.08E-03 | 1.94E-03 | -0.96 |  | 6.38E-03 | 6.79E-05 | 1.35 |  | 1.17E-04 | 3.14E-03 | 0.33 |  | 1.42E-01 | 7.19E-01 | 0.84 |  | 3.07E-02 | 1.29E-02 | -0.68 |
| Tyrosyl-Aspartate | 2.61 | 6.50E-04 | 1.72E-03 | 7.18E-04 | -0.61 |  | 1.52E-07 | 1.15E-07 | -1.16 |  | 3.54E-03 | 9.05E-04 | -0.59 |  | 3.75E-04 | 3.56E-06 | -0.54 |  | 6.09E-01 | 3.13E-01 | 0.14 |  | 1.21E-07 | 3.67E-02 | -1.11 |  | 3.48E-03 | 1.29E-02 | -0.53 |
| Uracil | 3.30 | 2.79E-10 | 2.67E-07 | 5.82E-10 | 7.00 |  | 1.54E-13 | 1.71E-09 | 6.62 |  | 3.16E-02 | 7.96E-02 | -0.40 |  | 1.96E-06 | 1.71E-09 | 7.36 |  | 8.70E-01 | 3.13E-01 | -0.16 |  | 1.69E-09 | 2.20E-06 | 6.88 |  | 8.08E-01 | 7.92E-01 | -0.12 |
| Urocanic acid | 3.78 | 2.30E-15 | 9.63E-07 | 1.53E-14 | 2.66 |  | 6.43E-06 | 5.90E-07 | -5.40 |  | 2.65E-18 | 3.53E-07 | -7.61 |  | 4.65E-01 | 1.27E-01 | -4.26 |  | 2.24E-03 | 1.11E-04 | -6.40 |  | 7.31E-02 | 3.82E-02 | 0.64 |  | 4.12E-06 | 2.21E-04 | -2.14 |
| Valyl-Leucine | 2.82 | 1.13E-06 | 4.02E-05 | 1.56E-06 | -0.88 |  | 8.30E-05 | 8.10E-04 | -0.88 |  | 9.79E-01 | 3.41E-01 | 0.00 |  | 1.63E-10 | 3.56E-06 | -1.21 |  | 9.50E-02 | 1.95E-01 | -0.25 |  | 1.58E-08 | 2.20E-06 | -0.93 |  | 5.84E-01 | 8.51E-01 | -0.05 |
| Vinylacetylglycine | 6.46 | 3.60E-08 | 2.67E-07 | 5.15E-08 | 9.08 |  | 1.60E-01 | 4.71E-01 | 0.54 |  | 9.78E-08 | 5.93E-07 | -8.26 |  | 4.60E-01 | 4.92E-01 | 0.28 |  | 8.49E-08 | 3.33E-09 | -8.62 |  | 4.21E-12 | 1.71E-09 | 5.67 |  | 3.44E-07 | 3.33E-09 | -3.63 |
| Xanthine | 4.73 | 2.99E-10 | 1.16E-06 | 6.06E-10 | -1.99 |  | 5.92E-08 | 1.66E-07 | -1.35 |  | 1.97E-02 | 1.08E-02 | 0.68 |  | 4.99E-09 | 6.86E-09 | -1.68 |  | 1.97E-01 | 2.64E-01 | 0.42 |  | 1.96E-10 | 1.73E-05 | -2.84 |  | 6.17E-01 | 7.92E-01 | -0.78 |
| Xanthurenic acid | 5.91 | 1.16E-15 | 9.63E-07 | 8.44E-15 | -2.89 |  | 6.67E-15 | 9.63E-07 | -2.41 |  | 2.98E-03 | 8.33E-03 | 0.52 |  | 8.19E-16 | 1.71E-09 | -5.74 |  | 5.44E-02 | 1.44E-03 | -2.23 |  | 6.31E-15 | 1.71E-09 | -2.40 |  | 2.44E-03 | 1.44E-03 | 0.52 |
| Trimethylamine | 2.98 | 1.41E-03 | 8.50E-05 | 1.49E-03 | -7.70 |  | 8.26E-01 | 4.38E-01 | 1.58 |  | 2.34E-06 | 4.87E-06 | 9.86 |  | 8.50E-01 | 3.95E-01 | -0.36 |  | 5.12E-07 | 1.13E-04 | 8.24 |  | 3.86E-02 | 2.85E-02 | -5.93 |  | 1.63E-01 | 2.32E-01 | 1.88 |
| ^a^Variable importance in the projection (VIP) was obtainedfrom the OPLS-DA model.^b^The p value was calculated from ANOVA. ^c^The p value was calculated fromnonparametric test Mann−Whitney U test. ^d^Fold change was calculated as a binarylogarithm of the average mass response (normalized peak area) ratio between each groupvscontrol group or between each  groupvsCKD group, where a positive value means that the average mass response of the metabolite in each group is larger than that inthe control group | | | | | | | | | | | | | | | | | | | | | | | | | | | | | |

Table S2. Differentially expressed metabolites between the Control and CKD model groups in week 6.

|  | CKD VS Control | | | | |  | EA VS Control | | |  | EA VS CKD | | |  | BU VS Control | | |  | BU VS CKD | | |  | PE VS Control | | |  | PE VS CKD | | |
| --- | --- | --- | --- | --- | --- | --- | --- | --- | --- | --- | --- | --- | --- | --- | --- | --- | --- | --- | --- | --- | --- | --- | --- | --- | --- | --- | --- | --- | --- |
| Metabolites | VIP | p^a^ | p^b^ | FDR^c^ | FC^d^ |  | p^a^ | p^b^ | FC^d^ |  | p^a^ | p^b^ | FC^d^ |  | p^a^ | p^b^ | FC^d^ |  | p^a^ | p^b^ | FC^d^ |  | p^a^ | p^b^ | FC^d^ |  | p^a^ | p^b^ | FC^d^ |
| 3-Methyldioxyindole | 12.53 | 1.90E-09 | 3.10E-06 | 1.78E-08 | 1.78 |  | 1.12E-07 | 1.79E-06 | 1.75 |  | 7.79E-02 | 1.19E-01 | -0.16 |  | 2.94E-09 | 3.10E-06 | 1.93 |  | 9.27E-01 | 8.13E-01 | -0.01 |  | 1.05E-06 | 5.25E-05 | 1.80 |  | 4.68E-01 | 3.85E-01 | -0.11 |
| Tryptophanol | 11.71 | 2.31E-06 | 4.67E-05 | 5.87E-06 | 1.05 |  | 9.73E-06 | 1.18E-05 | 0.94 |  | 4.88E-01 | 3.79E-01 | -0.10 |  | 2.15E-05 | 1.26E-04 | 0.88 |  | 1.93E-01 | 2.52E-01 | -0.15 |  | 2.22E-04 | 3.91E-04 | 0.92 |  | 7.86E-01 | 5.80E-01 | -0.12 |
| 8-Hydroxyguanine | 7.34 | 1.11E-09 | 1.72E-06 | 1.17E-08 | 7.53 |  | 7.20E-07 | 1.29E-08 | 6.98 |  | 2.79E-03 | 1.72E-03 | -0.82 |  | 6.38E-07 | 8.15E-05 | 6.36 |  | 4.57E-02 | 8.20E-02 | -3.03 |  | 1.31E-10 | 2.47E-06 | 13.44 |  | 7.12E-04 | 3.44E-03 | -0.74 |
| Creatinine | 7.08 | 6.79E-05 | 3.22E-04 | 1.16E-04 | -1.16 |  | 1.08E-03 | 2.31E-03 | -0.94 |  | 2.09E-01 | 2.02E-01 | 0.31 |  | 7.49E-06 | 2.34E-05 | -1.67 |  | 1.99E-01 | 1.79E-01 | -0.41 |  | 1.24E-05 | 3.67E-05 | -1.58 |  | 2.23E-01 | 1.79E-01 | -0.29 |
| 4,6-Dihydroxyquinoline | 6.18 | 5.63E-09 | 2.10E-06 | 3.38E-08 | 2.23 |  | 7.23E-04 | 2.31E-03 | 1.02 |  | 1.60E-06 | 1.26E-07 | -1.21 |  | 1.49E-08 | 3.32E-05 | 1.57 |  | 7.24E-02 | 2.20E-01 | -1.53 |  | 2.34E-05 | 6.26E-05 | 1.61 |  | 2.31E-03 | 7.19E-03 | -0.66 |
| Hydroxypyruvic acid | 5.08 | 2.62E-03 | 1.03E-06 | 3.23E-03 | -7.13 |  | 5.02E-03 | 4.81E-06 | -5.30 |  | 5.15E-02 | 1.52E-02 | 1.83 |  | 2.77E-03 | 3.11E-06 | -6.92 |  | 1.16E-01 | 2.95E-01 | 2.02 |  | 8.17E-03 | 2.17E-04 | -14.25 |  | 6.66E-02 | 4.02E-01 | 1.75 |
| 5-L-Glutamyl-taurine | 5.01 | 5.56E-03 | 1.03E-03 | 6.22E-03 | 1.06 |  | 3.41E-09 | 2.58E-08 | 1.52 |  | 3.11E-01 | 5.52E-03 | 0.30 |  | 2.89E-07 | 7.94E-06 | 1.25 |  | 8.29E-01 | 1.55E-01 | 0.11 |  | 7.94E-06 | 2.12E-05 | 1.46 |  | 3.47E-01 | 6.90E-02 | 0.25 |
| 3-Indole carboxylic acid glucuronide | 4.88 | 1.85E-03 | 3.12E-05 | 2.39E-03 | -5.86 |  | 2.92E-03 | 2.58E-08 | -4.27 |  | 4.74E-03 | 4.05E-02 | 1.50 |  | 1.96E-03 | 1.39E-03 | -4.94 |  | 2.69E-02 | 1.37E-02 | 5.73 |  | 2.67E-03 | 7.99E-04 | -10.82 |  | 9.13E-02 | 4.49E-02 | 3.85 |
| L-Fucose | 4.67 | 1.94E-08 | 9.55E-06 | 9.06E-08 | 1.68 |  | 1.43E-06 | 8.82E-06 | 1.63 |  | 4.14E-02 | 5.44E-02 | -0.21 |  | 3.86E-09 | 7.94E-06 | 1.92 |  | 6.56E-01 | 9.06E-01 | 0.06 |  | 2.97E-06 | 6.26E-05 | 1.71 |  | 3.64E-01 | 4.29E-01 | -0.13 |
| Pyridoxine | 4.30 | 3.71E-09 | 2.55E-06 | 2.59E-08 | 2.18 |  | 5.63E-05 | 1.55E-05 | 1.65 |  | 5.96E-01 | 3.79E-01 | -0.65 |  | 2.17E-04 | 1.41E-02 | 0.43 |  | 9.79E-01 | 4.29E-01 | -4.91 |  | 3.41E-05 | 1.21E-05 | 2.98 |  | 9.44E-01 | 6.35E-01 | -0.20 |
| Cysteine-S-sulfate | 4.15 | 2.25E-10 | 1.84E-06 | 3.77E-09 | 6.46 |  | 2.88E-02 | 1.55E-05 | 1.26 |  | 6.92E-08 | 6.46E-07 | -4.78 |  | 8.06E-03 | 1.10E-01 | 3.28 |  | 5.66E-01 | 1.41E-01 | -8.88 |  | 1.85E-02 | 1.75E-01 | 2.40 |  | 2.53E-04 | 7.27E-04 | -9.45 |
| Xanthosine | 4.08 | 2.31E-03 | 2.37E-04 | 2.94E-03 | 1.34 |  | 2.86E-10 | 1.29E-08 | 1.65 |  | 9.98E-01 | 8.56E-02 | 0.09 |  | 3.03E-05 | 2.34E-05 | 1.21 |  | 1.52E-01 | 2.06E-01 | -0.27 |  | 1.66E-04 | 2.05E-04 | 1.28 |  | 5.79E-01 | 7.52E-01 | -0.26 |
| Kynurenine | 4.04 | 4.73E-07 | 2.86E-06 | 1.42E-06 | -4.56 |  | 1.67E-06 | 3.87E-07 | -4.18 |  | 8.01E-01 | 9.22E-01 | -0.17 |  | 7.72E-07 | 1.41E-05 | -5.87 |  | 7.41E-01 | 3.56E-01 | -5.09 |  | 3.22E-04 | 5.30E-04 | -5.12 |  | 3.28E-02 | 4.99E-02 | 2.11 |
| L-Homocysteic acid | 4.02 | 1.16E-10 | 1.93E-06 | 3.25E-09 | 6.33 |  | 1.61E-02 | 2.67E-01 | 1.76 |  | 4.47E-08 | 1.26E-07 | -4.32 |  | 8.96E-03 | 3.40E-01 | 2.74 |  | 4.21E-01 | 1.75E-01 | -10.02 |  | 1.72E-02 | 2.85E-02 | 3.69 |  | 6.07E-05 | 4.23E-04 | -7.39 |
| Nicotinic acid | 4.00 | 1.99E-04 | 8.99E-04 | 3.03E-04 | -0.83 |  | 2.74E-04 | 9.97E-04 | -0.88 |  | 8.00E-01 | 6.26E-01 | 0.23 |  | 7.83E-06 | 1.26E-04 | -2.52 |  | 1.41E-01 | 7.52E-02 | -2.94 |  | 1.46E-07 | 6.26E-05 | -1.80 |  | 2.52E-03 | 7.19E-03 | -0.64 |
| 2-Hydroxyadipic acid | 3.93 | 1.84E-05 | 1.48E-04 | 3.86E-05 | 0.94 |  | 7.17E-06 | 5.80E-07 | 0.93 |  | 9.59E-01 | 5.20E-01 | -0.02 |  | 5.62E-06 | 7.72E-05 | 0.87 |  | 5.06E-01 | 3.63E-01 | -0.05 |  | 7.48E-05 | 1.05E-04 | 0.97 |  | 5.98E-01 | 8.74E-01 | 0.02 |
| Oleic acid | 3.83 | 2.60E-05 | 2.34E-05 | 5.20E-05 | -0.96 |  | 9.65E-04 | 9.97E-04 | -1.26 |  | 6.80E-01 | 7.70E-01 | -0.28 |  | 2.65E-03 | 3.44E-03 | -1.33 |  | 3.22E-01 | 1.79E-01 | -1.07 |  | 1.99E-02 | 6.49E-02 | -3.62 |  | 4.26E-01 | 9.37E-01 | -2.49 |
| 3-O-Methyldopa | 3.79 | 2.54E-04 | 3.22E-04 | 3.68E-04 | -1.36 |  | 6.50E-03 | 3.67E-03 | -0.59 |  | 2.03E-01 | 4.95E-01 | 0.79 |  | 7.17E-05 | 2.01E-04 | -2.64 |  | 3.73E-01 | 4.17E-01 | -2.65 |  | 2.53E-04 | 9.75E-04 | -2.41 |  | 5.61E-01 | 4.18E-01 | -0.07 |
| N-Acryloylglycine | 3.69 | 2.01E-08 | 6.13E-06 | 8.90E-08 | -4.96 |  | 1.21E-05 | 2.65E-05 | -5.43 |  | 2.97E-01 | 7.70E-01 | -0.05 |  | 5.62E-08 | 7.18E-06 | -6.39 |  | 8.00E-01 | 4.18E-01 | -2.74 |  | 4.67E-09 | 2.47E-06 | -12.08 |  | 5.02E-01 | 9.66E-01 | -0.77 |
| Succinyladenosine | 3.69 | 6.50E-08 | 3.18E-06 | 2.27E-07 | -6.89 |  | 3.12E-01 | 3.45E-01 | -2.19 |  | 3.24E-04 | 6.35E-04 | 4.32 |  | 6.61E-08 | 3.18E-06 | -7.33 |  | 7.72E-01 | 1.00E+00 | -0.06 |  | 7.13E-03 | 5.72E-03 | -8.82 |  | 1.51E-02 | 6.66E-03 | 7.44 |
| 4-Methoxycinnamic acid | 3.60 | 2.67E-11 | 2.10E-06 | 1.12E-09 | 2.48 |  | 1.16E-02 | 9.75E-02 | -0.42 |  | 1.12E-04 | 2.53E-04 | -2.76 |  | 1.02E-02 | 4.95E-02 | 0.01 |  | 8.96E-02 | 2.16E-02 | -6.47 |  | 5.05E-10 | 3.07E-06 | 3.27 |  | 1.23E-01 | 1.05E-01 | -0.32 |
| Acetylcysteine | 3.44 | 2.86E-03 | 7.19E-03 | 3.48E-03 | -0.42 |  | 5.45E-01 | 9.35E-01 | -0.01 |  | 2.97E-04 | 7.56E-04 | 1.46 |  | 1.11E-02 | 8.08E-03 | -1.90 |  | 6.15E-01 | 1.00E+00 | -1.02 |  | 3.01E-02 | 8.48E-02 | -5.76 |  | 2.99E-01 | 9.62E-02 | -3.08 |
| 5-Aminoimidazole | 3.38 | 1.67E-08 | 2.83E-06 | 8.26E-08 | -5.97 |  | 3.27E-07 | 5.80E-07 | -5.15 |  | 2.58E-01 | 2.81E-01 | 0.99 |  | 3.17E-09 | 1.03E-06 | -6.65 |  | 5.66E-01 | 9.59E-01 | -0.37 |  | 3.45E-09 | 1.13E-06 | -17.67 |  | 2.75E-01 | 3.90E-01 | -2.62 |
| Uric acid | 3.37 | 4.11E-03 | 2.68E-02 | 4.67E-03 | -1.48 |  | 3.14E-01 | 5.67E-01 | -0.31 |  | 3.12E-03 | 2.98E-02 | 1.48 |  | 7.46E-03 | 3.49E-03 | -5.04 |  | 7.61E-01 | 1.82E-01 | -8.32 |  | 6.76E-02 | 1.01E-01 | -4.32 |  | 2.57E-01 | 3.42E-01 | -0.62 |
| Sorbitol | 3.37 | 1.14E-06 | 2.00E-05 | 3.30E-06 | 3.88 |  | 6.88E-03 | 2.35E-02 | 1.53 |  | 1.91E-05 | 1.70E-04 | -2.28 |  | 5.92E-04 | 1.53E-03 | 2.18 |  | 3.00E-04 | 2.47E-03 | -3.45 |  | 1.35E-05 | 2.60E-04 | 5.64 |  | 3.11E-02 | 4.37E-02 | -1.47 |
| Deoxyadenosine | 3.33 | 1.01E-07 | 4.58E-06 | 3.40E-07 | -6.46 |  | 6.68E-02 | 7.42E-02 | -2.36 |  | 4.68E-04 | 1.06E-03 | 4.04 |  | 9.28E-08 | 1.28E-06 | -7.48 |  | 1.41E-01 | 1.38E-01 | -1.95 |  | 1.83E-03 | 3.38E-03 | -8.80 |  | 1.76E-02 | 1.34E-02 | 8.14 |
| Ethyl-N2-acetyl-L-argininate | 3.28 | 5.34E-06 | 4.67E-05 | 1.28E-05 | -0.87 |  | 3.26E-05 | 7.12E-05 | -1.29 |  | 5.05E-01 | 2.81E-01 | -0.11 |  | 5.39E-06 | 6.70E-05 | -4.83 |  | 1.51E-01 | 2.64E-01 | -9.72 |  | 8.30E-07 | 2.12E-05 | -1.28 |  | 2.83E-01 | 2.20E-01 | -0.16 |
| Valine | 3.23 | 6.86E-06 | 3.70E-05 | 1.60E-05 | -3.28 |  | 1.07E-05 | 2.65E-05 | -2.93 |  | 7.59E-01 | 4.95E-01 | 1.27 |  | 7.10E-05 | 3.20E-04 | -2.59 |  | 2.50E-01 | 1.74E-01 | 4.21 |  | 1.94E-05 | 9.52E-05 | -10.37 |  | 6.84E-01 | 5.11E-01 | -0.76 |
| Adrenoylethanolamide | 3.20 | 2.15E-05 | 5.79E-04 | 4.41E-05 | 5.23 |  | 7.49E-02 | 2.09E-02 | 2.89 |  | 5.20E-03 | 2.40E-02 | -2.45 |  | 1.13E-02 | 6.81E-06 | 6.66 |  | 4.15E-01 | 1.00E+00 | 3.28 |  | 1.33E-01 | 3.12E-02 | 6.26 |  | 6.50E-01 | 4.32E-02 | -4.51 |
| Homoagmatine | 3.17 | 1.32E-06 | 3.32E-05 | 3.57E-06 | 1.23 |  | 2.81E-07 | 2.45E-07 | 1.15 |  | 4.90E-01 | 3.18E-01 | -0.08 |  | 1.69E-02 | 9.08E-03 | -0.12 |  | 2.84E-02 | 3.98E-02 | -2.83 |  | 8.59E-06 | 4.40E-05 | 1.12 |  | 5.64E-01 | 4.77E-01 | -0.11 |
| Trimethylamine | 3.15 | 3.39E-03 | 1.53E-05 | 3.96E-03 | -6.03 |  | 6.06E-03 | 3.23E-04 | -5.63 |  | 2.33E-01 | 5.45E-01 | 0.64 |  | 4.28E-03 | 1.40E-04 | -5.39 |  | 1.05E-01 | 8.31E-02 | 3.27 |  | 1.12E-02 | 2.54E-03 | -11.05 |  | 6.21E-02 | 2.99E-02 | 4.59 |
| Valyl-Cysteine | 3.11 | 1.80E-10 | 2.05E-06 | 3.78E-09 | 3.42 |  | 1.38E-02 | 1.61E-01 | 0.30 |  | 2.13E-04 | 5.32E-04 | -2.93 |  | 1.19E-03 | 2.63E-04 | 2.39 |  | 1.93E-01 | 2.42E-02 | -2.84 |  | 4.89E-06 | 3.16E-04 | 4.12 |  | 1.14E-01 | 1.55E-01 | -2.66 |
| Tyramine | 3.10 | 1.02E-04 | 4.66E-05 | 1.71E-04 | 4.65 |  | 4.36E-01 | 4.86E-01 | -0.56 |  | 2.86E-04 | 7.34E-05 | -4.44 |  | 5.42E-01 | 6.07E-01 | -0.36 |  | 1.76E-04 | 7.98E-05 | -13.43 |  | 1.39E-04 | 1.51E-03 | 8.57 |  | 7.63E-02 | 1.65E-01 | -2.34 |
| Methyladenine | 2.98 | 3.98E-08 | 4.59E-06 | 1.59E-07 | -6.38 |  | 1.06E-06 | 5.62E-05 | -5.35 |  | 1.73E-01 | 1.19E-01 | 1.09 |  | 4.08E-08 | 8.42E-06 | -6.67 |  | 3.41E-01 | 3.33E-01 | 0.88 |  | 1.05E-07 | 1.39E-05 | -16.02 |  | 3.10E-01 | 3.02E-01 | 1.02 |
| 3-Oxohexanoic acid | 2.93 | 1.58E-04 | 2.09E-06 | 2.60E-04 | -6.45 |  | 6.93E-01 | 6.53E-01 | -1.68 |  | 3.13E-04 | 5.32E-04 | 4.31 |  | 2.72E-04 | 7.61E-06 | -5.92 |  | 2.28E-01 | 1.75E-01 | 3.17 |  | 1.10E-01 | 2.55E-02 | -6.83 |  | 7.46E-03 | 1.02E-03 | 9.66 |
| L-Homoserine | 2.83 | 1.29E-06 | 1.36E-05 | 3.60E-06 | 2.46 |  | 1.18E-01 | 1.87E-01 | 0.13 |  | 1.59E-03 | 4.82E-03 | -2.17 |  | 3.89E-01 | 1.51E-01 | -2.64 |  | 3.61E-06 | 4.12E-05 | -12.78 |  | 1.01E-01 | 1.89E-01 | -0.60 |  | 2.16E-03 | 7.13E-03 | -5.31 |
| 4-Aminohippuric acid | 2.71 | 3.70E-05 | 3.22E-04 | 6.91E-05 | -0.71 |  | 3.15E-07 | 3.87E-07 | -4.08 |  | 4.75E-02 | 1.29E-01 | -1.96 |  | 1.53E-04 | 1.86E-04 | -4.33 |  | 4.02E-01 | 5.83E-02 | -7.05 |  | 6.14E-03 | 1.71E-02 | -3.44 |  | 2.74E-01 | 3.04E-01 | -0.86 |
| 3-Methylhistidine | 2.69 | 4.48E-05 | 2.33E-04 | 8.01E-05 | -1.87 |  | 4.67E-05 | 2.75E-04 | -3.26 |  | 8.93E-01 | 1.00E+00 | -0.07 |  | 2.74E-07 | 1.51E-05 | -6.07 |  | 9.01E-02 | 3.59E-02 | -8.12 |  | 7.57E-09 | 8.22E-06 | -11.63 |  | 1.02E-02 | 2.26E-02 | -4.79 |
| Valyl-Leucine | 2.61 | 3.55E-05 | 2.75E-04 | 6.77E-05 | -2.22 |  | 5.93E-04 | 1.74E-03 | -1.61 |  | 3.38E-01 | 3.18E-01 | 0.71 |  | 5.25E-07 | 1.58E-05 | -3.36 |  | 2.03E-01 | 1.69E-01 | -2.38 |  | 6.17E-08 | 8.16E-06 | -5.22 |  | 5.16E-02 | 2.50E-02 | -0.23 |
| Galactonic acid | 2.60 | 1.82E-07 | 1.04E-04 | 5.66E-07 | 4.00 |  | 4.68E-01 | 2.61E-01 | -0.64 |  | 2.47E-05 | 3.06E-04 | -4.15 |  | 9.06E-01 | 9.65E-03 | -1.60 |  | 1.30E-06 | 3.30E-05 | -15.58 |  | 2.68E-02 | 3.91E-02 | 4.56 |  | 9.34E-04 | 4.86E-03 | -3.43 |
| Tyrosyl-Threonine | 2.57 | 8.90E-06 | 1.72E-06 | 2.02E-05 | 5.32 |  | 3.38E-01 | 6.13E-01 | -0.12 |  | 3.11E-05 | 4.46E-07 | -4.97 |  | 1.48E-01 | 1.00E+00 | 0.35 |  | 1.01E-04 | 3.91E-05 | -14.46 |  | 2.40E-02 | 1.28E-01 | 3.30 |  | 1.14E-03 | 4.51E-04 | -9.87 |
| Histidinyl-Tyrosine | 2.53 | 1.91E-02 | 4.38E-02 | 1.98E-02 | 0.65 |  | 7.19E-10 | 3.07E-06 | 1.84 |  | 1.66E-04 | 6.35E-04 | 0.85 |  | 7.59E-08 | 1.15E-05 | 1.65 |  | 5.86E-03 | 6.38E-03 | 0.80 |  | 2.56E-06 | 1.46E-05 | 1.48 |  | 3.76E-02 | 6.90E-02 | 0.51 |
| Methylcytosine | 2.52 | 3.61E-03 | 8.08E-03 | 4.16E-03 | -1.85 |  | 1.40E-03 | 2.25E-03 | -3.31 |  | 5.37E-01 | 4.95E-01 | -0.70 |  | 2.39E-04 | 8.77E-05 | -6.06 |  | 1.53E-01 | 1.54E-02 | -10.40 |  | 5.00E-05 | 6.63E-06 | -6.84 |  | 1.23E-02 | 6.23E-02 | -2.59 |
| Histamine | 2.47 | 3.61E-04 | 3.20E-04 | 4.97E-04 | -4.20 |  | 1.31E-01 | 1.15E-01 | -3.71 |  | 1.13E-02 | 3.38E-02 | 1.04 |  | 2.10E-02 | 3.10E-02 | -1.61 |  | 5.82E-02 | 2.39E-02 | 8.99 |  | 1.37E-04 | 2.16E-04 | -14.75 |  | 5.28E-01 | 3.49E-01 | -1.98 |
| Deoxyribose | 2.47 | 8.86E-10 | 3.20E-04 | 1.06E-08 | -3.63 |  | 5.12E-03 | 6.57E-03 | -2.07 |  | 1.84E-03 | 1.20E-02 | 1.55 |  | 1.25E-10 | 1.83E-06 | -5.67 |  | 1.42E-01 | 7.62E-02 | -5.00 |  | 7.54E-04 | 2.00E-03 | -2.11 |  | 2.37E-04 | 2.26E-04 | 5.67 |
| Tyrosyl-Aspartate | 2.46 | 3.45E-04 | 1.79E-03 | 4.84E-04 | 0.65 |  | 4.75E-08 | 3.07E-06 | 1.08 |  | 2.79E-04 | 7.56E-04 | 0.39 |  | 1.02E-03 | 2.34E-03 | 0.74 |  | 3.87E-01 | 3.63E-01 | 0.07 |  | 6.07E-04 | 8.41E-04 | 0.81 |  | 2.46E-01 | 5.27E-01 | 0.14 |
| 2-Hydroxycinnamic acid | 2.42 | 2.44E-08 | 3.10E-06 | 1.02E-07 | 1.19 |  | 2.68E-01 | 3.03E-01 | -3.56 |  | 1.16E-06 | 1.37E-05 | -4.15 |  | 2.88E-01 | 1.00E+00 | -2.16 |  | 3.05E-02 | 1.71E-02 | -8.73 |  | 1.67E-02 | 1.21E-02 | -2.78 |  | 3.68E-02 | 6.31E-02 | -3.93 |
| Methylhippuric acid | 2.36 | 7.10E-10 | 6.65E-09 | 9.94E-09 | -2.86 |  | 3.27E-10 | 3.44E-06 | -4.02 |  | 3.38E-01 | 3.18E-01 | -0.90 |  | 4.73E-11 | 1.93E-06 | -3.92 |  | 2.95E-01 | 3.17E-01 | -3.18 |  | 3.00E-08 | 4.36E-06 | -7.54 |  | 9.31E-01 | 7.35E-01 | -1.99 |
| L-Canaline | 2.36 | 2.21E-09 | 1.26E-07 | 1.69E-08 | -5.70 |  | 2.66E-06 | 4.25E-05 | -5.61 |  | 3.57E-01 | 6.82E-01 | 0.39 |  | 3.36E-02 | 1.49E-03 | -5.09 |  | 1.60E-01 | 3.59E-01 | 2.42 |  | 1.07E-06 | 4.05E-05 | -14.00 |  | 3.11E-01 | 3.30E-01 | 2.12 |
| Indoxyl | 2.35 | 7.09E-04 | 1.25E-03 | 9.45E-04 | 1.86 |  | 2.62E-03 | 3.45E-03 | 1.39 |  | 4.13E-03 | 1.52E-02 | -0.44 |  | 3.01E-01 | 2.59E-01 | 0.21 |  | 1.47E-03 | 4.92E-03 | -2.33 |  | 8.24E-03 | 2.14E-03 | 1.50 |  | 3.63E-02 | 1.33E-01 | 0.62 |
| 1-Phenylethylamine | 2.31 | 7.24E-04 | 1.47E-03 | 9.50E-04 | -3.09 |  | 4.99E-03 | 6.84E-03 | -4.13 |  | 5.96E-01 | 8.00E-01 | 0.16 |  | 2.58E-04 | 6.27E-05 | -5.85 |  | 5.19E-01 | 1.53E-01 | -5.15 |  | 7.32E-03 | 3.58E-02 | -4.64 |  | 2.83E-01 | 2.54E-01 | 5.25 |
| Hypotaurine | 2.27 | 6.26E-08 | 1.81E-06 | 2.29E-07 | -4.20 |  | 4.57E-08 | 1.30E-05 | -4.99 |  | 6.75E-01 | 8.61E-01 | -0.41 |  | 5.21E-09 | 3.18E-06 | -6.04 |  | 1.47E-01 | 1.23E-01 | -4.69 |  | 2.14E-08 | 1.30E-05 | -12.38 |  | 3.73E-01 | 6.78E-01 | -1.77 |
| Acetoacetic acid | 2.26 | 2.02E-04 | 1.33E-08 | 3.03E-04 | -6.12 |  | 5.11E-01 | 2.70E-01 | -2.38 |  | 1.47E-03 | 5.52E-03 | 3.43 |  | 2.32E-04 | 7.99E-07 | -6.30 |  | 4.79E-01 | 5.90E-01 | 0.93 |  | 1.65E-01 | 2.45E-02 | -10.88 |  | 1.27E-02 | 1.89E-02 | 6.26 |
| 1-Methylxanthine | 2.26 | 1.05E-07 | 1.33E-08 | 3.39E-07 | 1.14 |  | 8.64E-01 | 7.87E-01 | -2.14 |  | 6.24E-05 | 3.71E-05 | -2.91 |  | 6.26E-01 | 2.28E-01 | -3.35 |  | 4.03E-02 | 2.70E-02 | -12.09 |  | 2.46E-01 | 1.01E-01 | -4.20 |  | 1.01E-03 | 3.41E-03 | -5.19 |
| Deoxyuridine | 2.25 | 3.39E-12 | 1.33E-08 | 2.85E-10 | -5.59 |  | 2.95E-05 | 1.55E-04 | -5.47 |  | 1.56E-03 | 2.72E-03 | -3.26 |  | 2.75E-02 | 2.36E-02 | -5.25 |  | 8.80E-02 | 7.12E-02 | -9.58 |  | 1.31E-02 | 1.24E-02 | -11.93 |  | 4.75E-02 | 3.97E-02 | -7.34 |
| Hippuric acid | 2.24 | 2.05E-04 | 3.71E-05 | 3.03E-04 | -3.07 |  | 5.30E-05 | 2.27E-06 | -6.59 |  | 3.57E-03 | 6.32E-03 | -2.05 |  | 3.13E-05 | 1.28E-06 | -6.59 |  | 3.71E-03 | 1.01E-03 | -9.12 |  | 8.88E-05 | 1.82E-05 | -13.37 |  | 5.25E-02 | 8.17E-02 | -4.04 |
| L-Phenylalanine | 2.23 | 4.03E-05 | 1.39E-04 | 7.37E-05 | -2.12 |  | 8.39E-06 | 7.40E-05 | -2.46 |  | 4.92E-01 | 3.38E-01 | -0.02 |  | 9.01E-07 | 5.34E-05 | -3.36 |  | 2.14E-01 | 1.35E-01 | -2.40 |  | 3.97E-08 | 1.73E-05 | -6.37 |  | 2.79E-02 | 1.90E-02 | -1.31 |
| 2-Octenoylcarnitine | 2.23 | 4.02E-09 | 1.37E-05 | 2.60E-08 | -4.76 |  | 4.93E-02 | 5.81E-02 | -1.47 |  | 2.61E-04 | 1.72E-03 | 2.94 |  | 9.66E-06 | 2.81E-03 | -1.91 |  | 8.48E-05 | 3.51E-04 | 9.76 |  | 5.02E-08 | 3.56E-05 | -12.95 |  | 5.14E-01 | 8.64E-01 | -0.10 |
| 12-Methyltridecanoic acid | 2.22 | 3.16E-03 | 1.25E-03 | 3.79E-03 | 2.28 |  | 2.82E-02 | 1.07E-02 | 1.38 |  | 3.23E-01 | 1.19E-01 | -0.05 |  | 5.61E-04 | 2.97E-02 | 1.38 |  | 2.91E-01 | 1.43E-01 | -0.71 |  | 5.38E-03 | 4.79E-03 | 1.22 |  | 5.11E-01 | 4.18E-01 | -0.09 |
| Trimethylamine-N-oxide | 2.19 | 1.40E-02 | 2.01E-03 | 1.49E-02 | 0.30 |  | 9.94E-01 | 9.50E-01 | -0.03 |  | 1.81E-02 | 4.94E-02 | -0.44 |  | 6.75E-02 | 4.35E-04 | -0.14 |  | 8.39E-01 | 7.82E-01 | -1.30 |  | 1.57E-02 | 7.47E-03 | 0.39 |  | 9.27E-01 | 8.43E-01 | -0.04 |
| Aminoadipic acid | 2.12 | 1.15E-08 | 4.46E-07 | 6.02E-08 | -5.48 |  | 7.11E-01 | 2.00E-01 | -2.75 |  | 7.14E-03 | 4.05E-02 | 2.55 |  | 4.30E-07 | 2.09E-04 | -4.79 |  | 1.06E-01 | 1.39E-01 | 3.25 |  | 1.07E-05 | 9.02E-05 | -13.63 |  | 1.14E-01 | 2.22E-01 | 2.30 |
| 2-Pyrrolidinone | 2.11 | 1.92E-04 | 4.44E-04 | 2.98E-04 | -2.17 |  | 1.68E-04 | 7.23E-04 | -1.84 |  | 9.66E-01 | 9.22E-01 | -0.31 |  | 2.71E-06 | 8.17E-03 | -4.39 |  | 3.58E-02 | 2.44E-02 | -6.91 |  | 2.42E-05 | 1.74E-04 | -2.79 |  | 3.58E-01 | 3.13E-01 | -0.41 |
| Homocysteine | 2.10 | 5.54E-08 | 4.19E-06 | 2.11E-07 | -3.30 |  | 6.33E-03 | 4.01E-02 | -0.58 |  | 8.57E-02 | 5.20E-01 | -0.15 |  | 5.08E-03 | 5.66E-03 | -0.70 |  | 1.22E-01 | 3.23E-01 | -0.35 |  | 4.43E-04 | 5.11E-03 | -2.53 |  | 4.38E-03 | 9.08E-03 | -1.98 |
| Vinylacetylglycine | 2.04 | 2.73E-05 | 7.72E-05 | 5.33E-05 | 1.41 |  | 5.05E-01 | 3.71E-01 | -1.10 |  | 2.55E-02 | 1.35E-02 | -2.02 |  | 4.36E-03 | 2.55E-04 | -4.51 |  | 1.95E-07 | 1.32E-05 | -14.72 |  | 4.93E-02 | 1.90E-02 | -0.70 |  | 5.71E-02 | 5.81E-02 | -2.62 |
| Indole | 2.03 | 7.67E-03 | 5.66E-03 | 8.48E-03 | 0.29 |  | 3.20E-01 | 3.71E-01 | 1.75 |  | 3.02E-03 | 3.25E-03 | -0.16 |  | 2.94E-09 | 1.48E-04 | 1.93 |  | 9.27E-01 | 8.13E-01 | -0.01 |  | 1.05E-06 | 5.25E-05 | 1.80 |  | 4.68E-01 | 3.85E-01 | -0.11 |
| Tryptophan | 2.00 | 1.63E-04 | 7.41E-05 | 2.63E-04 | -4.39 |  | 4.13E-02 | 1.10E-01 | -1.94 |  | 2.30E-02 | 2.67E-02 | 3.10 |  | 1.64E-06 | 3.62E-01 | -5.65 |  | 4.33E-01 | 7.58E-01 | -0.52 |  | 3.07E-04 | 4.72E-04 | -10.30 |  | 6.58E-01 | 4.44E-01 | 4.42 |
| N-Acetylleucine | 1.97 | 2.72E-04 | 1.34E-03 | 3.87E-04 | -2.17 |  | 1.34E-06 | 1.30E-05 | -5.50 |  | 2.81E-02 | 2.98E-02 | -1.76 |  | 2.22E-08 | 5.43E-04 | -6.79 |  | 8.21E-04 | 4.86E-04 | -10.89 |  | 1.18E-05 | 7.77E-05 | -12.92 |  | 1.49E-01 | 5.35E-02 | -5.09 |
| L-Serine | 1.96 | 9.28E-09 | 7.78E-06 | 5.20E-08 | -2.96 |  | 1.49E-06 | 5.21E-05 | -2.53 |  | 2.03E-01 | 3.58E-01 | 0.63 |  | 1.13E-08 | 6.50E-05 | -4.30 |  | 5.05E-01 | 2.22E-01 | -3.92 |  | 2.61E-06 | 7.40E-05 | -4.79 |  | 1.69E-01 | 3.01E-01 | 1.75 |
| Threoninyl-Proline | 1.94 | 2.11E-09 | 3.03E-06 | 1.78E-08 | -2.39 |  | 9.66E-09 | 6.77E-06 | -4.72 |  | 4.78E-01 | 3.18E-01 | -0.99 |  | 3.47E-12 | 1.28E-03 | -5.30 |  | 8.13E-04 | 2.82E-03 | -6.39 |  | 4.08E-06 | 5.24E-05 | -3.68 |  | 6.26E-02 | 9.57E-02 | 2.83 |
| Phenylacetylglycine | 1.93 | 3.19E-03 | 4.43E-03 | 3.77E-03 | 0.48 |  | 8.38E-01 | 7.56E-01 | -1.31 |  | 4.06E-02 | 4.84E-02 | -1.62 |  | 6.86E-02 | 6.38E-03 | -0.77 |  | 8.75E-01 | 8.43E-01 | -3.59 |  | 1.10E-02 | 8.44E-03 | -0.67 |  | 9.79E-01 | 7.82E-01 | -1.19 |
| Methylxanthine | 1.91 | 2.82E-06 | 2.10E-06 | 6.97E-06 | 2.44 |  | 8.64E-01 | 7.87E-01 | -2.14 |  | 6.24E-05 | 3.71E-05 | -2.91 |  | 6.26E-01 | 5.01E-01 | -3.35 |  | 5.03E-02 | 2.70E-02 | -12.09 |  | 2.46E-01 | 1.01E-01 | -4.20 |  | 1.01E-03 | 3.41E-03 | -5.19 |
| Pyrimidine | 1.90 | 3.77E-04 | 6.51E-03 | 5.10E-04 | 1.59 |  | 1.07E-02 | 4.25E-02 | 1.12 |  | 1.87E-01 | 2.02E-01 | -0.56 |  | 3.11E-01 | 3.75E-02 | -1.15 |  | 1.49E-02 | 1.88E-02 | -8.04 |  | 1.61E-04 | 1.69E-03 | 4.21 |  | 2.41E-01 | 2.66E-01 | 1.76 |
| Histine | 1.90 | 1.78E-06 | 2.81E-05 | 4.67E-06 | -4.23 |  | 5.64E-08 | 1.37E-05 | -5.10 |  | 5.78E-01 | 7.11E-01 | -0.48 |  | 5.99E-06 | 1.24E-02 | -5.05 |  | 8.97E-01 | 6.86E-01 | -1.37 |  | 1.89E-02 | 1.39E-02 | -8.73 |  | 1.33E-01 | 1.66E-01 | 3.26 |
| p-Cresol | 1.89 | 1.80E-04 | 6.29E-04 | 2.85E-04 | -2.67 |  | 4.10E-04 | 7.14E-04 | -2.51 |  | 8.55E-01 | 1.00E+00 | 1.04 |  | 5.86E-08 | 1.02E-02 | -5.19 |  | 1.16E-01 | 1.18E-01 | -4.21 |  | 1.34E-06 | 3.28E-05 | -9.46 |  | 2.76E-01 | 3.73E-01 | 0.27 |
| ^a^Variable importance in the projection (VIP) was obtained from the OPLS-DA model. ^b^The p value was calculated from ANOVA. ^c^The p value was calculated from nonparametric test Mann−Whitney U test. ^d^Fold change was calculated as a binary logarithm of the average mass response (normalized peak area) ratio between each group vs control group or between each group vs CKD group, where a positive value means that the average mass response of the metabolite in each group is larger than that in the control group | | | | | | | | | | | | | | | | | | | | | | | | | | | | | |

Figure S1


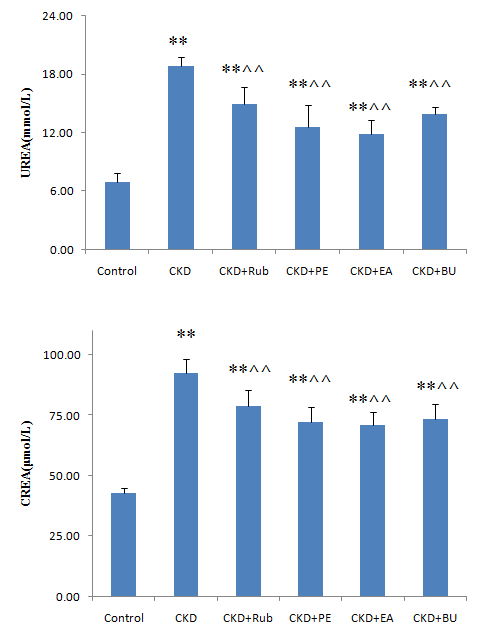


Figure S1. Biochemical parameters in the normal control, CKD, CKD+Rub, CKD+EA, CKD+BU and CKD+PE groups. Creatinine (CREA) and urea in the control, untreated CKD, Rub-treated CKD (CKD+Rub), EA-treated CKD (CKD+EA), BU-treated CKD (CKD+BU) and PE-treated CKD (CKD+PE) groups. *p < 0.05, **p < 0.01 compared to control group; ^p < 0.05, ^^p < 0.01 compared to CKD group.

Figure S2


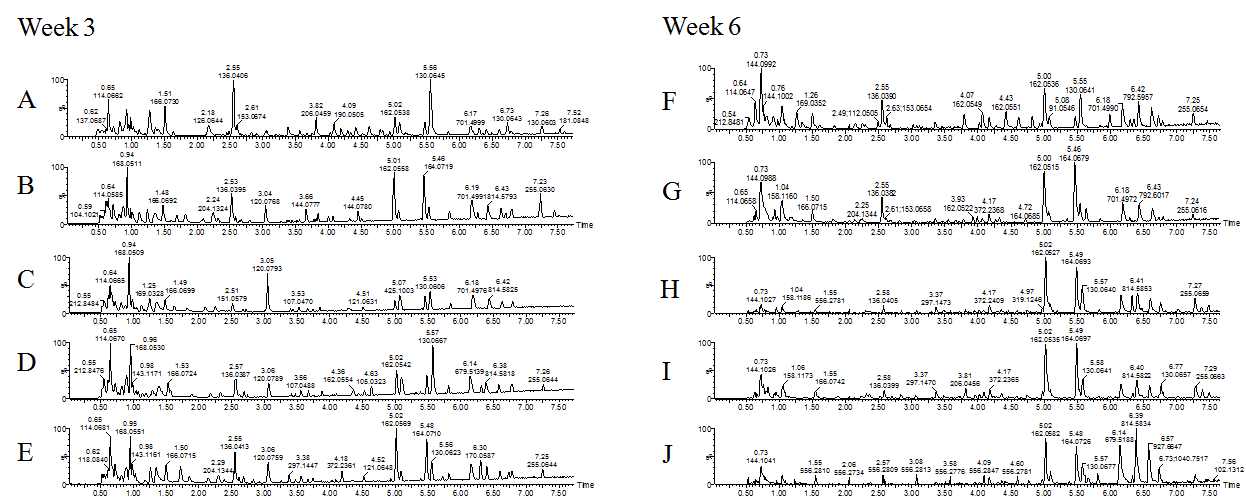


Figure S2. The representative BPI chromatograms of urine samples from each group in week 3 and week 6. (A) Control group, (B) CKD group, (C) CKD+PE group, (D) CKD+EA, (E) CKD+BU; (F) Control group, (G) CKD group, (H) CKD+PE group, (I) CKD+EA, (J) CKD+BU.
